# The decline in adolescent substance use across Europe and North America in the early twenty-first century: A result of the digital revolution?

**DOI:** 10.1007/s00038-018-1182-7

**Published:** 2018-12-17

**Authors:** Margaretha De Looze, S. van Dorsselaer, G. W. J. M. Stevens, M. Boniel-Nissim, A. Vieno, R. J. J. M. Van den Eijnden

**Affiliations:** 1 Department of Interdisciplinary Social Science, Faculty of Social and Behavioural Sciences, Utrecht University, P.O. Box 80.140, 3508 TC Utrecht, The Netherlands; 2 Netherlands Institute of Mental Health and Addiction, Utrecht, The Netherlands; 3 Department of Behavioral Sciences, School of Social Sciences and Humanities, Kinneret Academic College, Sea of Galilee, Israel; 4 Department of Developmental and Social Psychology, University of Padova, Padova, Italy

**Keywords:** Adolescence, Substance use, Tobacco, Alcohol, Cannabis, Electronic media communication, Internet, Trends over time, Europe, Time spent with friends

## Abstract

**Objectives:**

Increases in electronic media communication (EMC) and decreases in face-to-face peer contact in the evening (FTF) have been thought to explain the recent decline in adolescent substance use (alcohol, tobacco, cannabis). This study addresses this hypothesis, by examining associations between (time trends in) EMC, FTF, and substance use in more than 25 mainly European countries.

**Methods:**

Using 2002–2014 data from the international Health Behaviour in School-aged Children (HBSC) study, we ran multilevel logistic regression analyses to investigate the above associations.

**Results:**

National declines in substance use were associated with declines in FTF, but not with increases in EMC. At the individual level, both EMC and FTF related positively to substance use. For alcohol and cannabis use, the positive association with EMC was stronger in more recent years. Associations between EMC and substance use varied across countries, but this variation could not be explained by the proportion of young people using EMC within countries.

**Conclusions:**

Our research suggests that the decrease in FTF, but not the increase in EMC, plays a role in the recent decrease in adolescent substance use.

**Electronic supplementary material:**

The online version of this article (10.1007/s00038-018-1182-7) contains supplementary material, which is available to authorized users.

## Introduction

Since the early 2000s, two impactful parallel transitions have taken place in the daily lives of adolescents across Europe and North America. First, substance (tobacco, alcohol, cannabis) use decreased substantially (De Looze et al. 2015; Hublet et al. 2015). To illustrate, data from the international Health Behaviour in School-aged Children (HBSC) study showed that the percentage of 15 year olds in Europe and North America who smoke weekly decreased from 24% in 2002 to 12% in 2014. Weekly alcohol use decreased from 29% to 13%, and lifetime cannabis use among 15 year olds decreased from 22% to 15% (Currie et al. 2004; Inchley et al. 2016). The decline in substance use took place across virtually all countries and regions included in the study. At the same time, the so-called digital revolution took place. The proportion of households with children that had home access to Internet increased substantially (Eurostat 2018), and rates of mobile and smart phone usage among teenagers expended enormously (Haddon et al. 2012). With the rise of social networking sites and instant messenger functions, electronic media communication (EMC) became an integral part of adolescents’ daily lives (Boniel-Nissim et al. 2015a).

Some researchers suggested that the increase in EMC in the early twenty-first century can partly explain the decline in adolescent substance use (e.g. Livingston 2014; Twenge 2017). Specifically, they propose that EMC reduces the time adolescents spend face-to-face (FTF) with peers. The idea that EMC partly replaces adolescents’ FTF time with peers has been referred to as the *displacement* hypothesis (e.g. Kraut et al. 1998; Mesch 2003; Nie 2001). As substance use typically takes place in contexts where young people socialize in person, for example, in bars and pubs at night, the displacement hypothesis poses that an increase in EMC not only leads to a decrease in FTF, but also to a decrease in substance use. While the displacement hypothesis predicts that increases over time in EMC are associated with decreases over time in FTF and substance use, the *stimulation* hypothesis (Valkenburg and Peter 2007a) suggests the opposite. It proposes that adolescents who are active users of EMC, spend *more* face-to-face time with their friends, because Internet-based communication technologies encourage communication with existing friends (e.g. Bryant et al. 2006; Kuntsche et al. 2009; Valkenburg and Peter 2007a, b, 2011). Adolescents may, for example, use EMC to communicate with peers about where and when to meet and what to do offline (Kuntsche et al. 2009; Subrahmanyam and Greenfield 2008). As a consequence, EMC may be associated with *more* rather than less substance use.

In sum, both the displacement and stimulation hypothesis pose that electronic media communication may affect adolescents’ substance use through its influence on their time spent with friends. However, the displacement hypothesis assumes a negative effect from online communication to time spent with existing friends, whereas the stimulation hypothesis predicts a positive relationship between these variables. While early research in the field of social media use appears to confirm the displacement hypothesis (Kraut et al. 1998; Nie 2001; Nie et al. 2002; Weiser 2001), more recent research provides support for the stimulation hypothesis (Gommans et al. 2015; Kraut et al. 2002; Valkenburg and Peter 2007a, b, 2011).

A potential explanation for these inconsistent findings is that the association between EMC, FTF and substance use depends on the context (i.e. time and country) in which studies are conducted. Possibilities for communicating electronically vary considerably across countries and time. To illustrate, in Denmark in 2010, 98% of the children aged 9–16 had Internet access at home and 75% had profiles on social networking sites. In Romania, these percentages were considerably lower (85% and 46%; Haddon et al. 2012). Moreover, the percentage of European youth who had access to the Internet increased significantly over time, from 67% to 96% between 2007 and 2016 (Eurostat 2018). Based on the assumption that EMC more likely functions as a stimulator of social connections in contexts where the majority of young people uses EMC, we pose that EMC relates positively to FTF in contexts where EMC is highly available to young people (i.e. in countries and time periods with high rates of EMC use). If many friends are online, young people may be more likely to use EMC to communicate with existing friends. In contrast, in contexts where a considerable part of young people is not online (yet), EMC may function more as a displacement of FTF. As such, we hypothesized that in the early 2000s and in countries where EMC is less available, EMC was associated with low FTF and consequently, low substance use. In contrast, in more recent years and in countries where large proportions of youth use EMC, we expected EMC to be positively associated with FTF and, consequently, with substance use.

### The present study

This study analyses trends over time in adolescent substance use, FTF and EMC and assesses whether and how these behaviours are associated across Europe, Canada and Israel in the period 2002–2014. Using data from the international Health Behaviour in School-aged Children (HBSC) study, we aimed to answer the following research questions:

1. Are country-level trends over time in substance use associated with country-level trends over time in FTF and EMC?
2. Have associations between substance use and EMC changed across time?
3. Do associations between substance use and EMC differ across countries?

The first two research questions were analysed based on data from 2002, 2006 and 2010. Due to the introduction of new measures for EMC and FTF in 2014, the 2014 data could not be included in the trend analyses and were analysed separately. In line with recent research (Gommans et al. 2015; Kraut et al. 2002; Valkenburg and Peter 2007a, b, 2011), we overall expected to find support for the stimulation hypothesis, stating that EMC relates positively to FTF and substance use. However, we predicted that these associations varied across time and country, with more positive associations in more recent years and in countries with large proportions of youth using EMC.

## Method

### Study design

Data were obtained from the WHO collaborative HBSC study conducted in 2002–2014. HBSC is an international survey on adolescent (11-, 13-, and 15 year olds) health and the context of health, conducted every 4 years since 1983 across European and North American countries (Inchley et al. 2016). Sampling procedure and questionnaire are based on a standardized research protocol (Currie et al. 2014). Each participating country obtained approval to conduct the survey from their ethics review board or equivalent regulatory institution. Data were collected through a school-based survey, using an anonymous self-completion questionnaire. Under supervision of the national research teams, a translation/back-translation procedure was applied to guarantee language equivalence of the questionnaires. Representative samples on each national or regional level were selected using a clustered sampling design where the initial sampling unit was either the class or the school.

### Participants

Thirty HBSC countries collected data on EMC, FTF and substance use in 2002, 2006, and 2010. Separate studies were carried out in different parts of Belgium (Flanders and Wallonia), and the UK (England, Scotland and Wales). The data for these countries were analysed at the national level. Danish data were based on mainland Denmark (data from Greenland were excluded from the analyses). In total, the analyses for tobacco and alcohol use were based on *N* = 445,827 adolescents (51% girls, *M*_age_ = 13.5 years old) across 26 countries. As cannabis use was assessed in 25 countries and only among 15 year olds, analyses on cannabis use were based on *N* = 137,398.

In 2014, 42 HBSC countries participated in the HBSC study. Slovakia and Lithuania did not have (reliable) data on EMC; Spain and Ireland lacked data on FTF. These countries were removed from the analysis. This resulted in a sample of *N*=191,727 adolescents in 34 countries. The mean age of adolescents was 13.5 years old (ranging from 13.1 in Armenia to 13.8 in Canada). 51% were girls (ranging from 44% in Russia to 53% in Austria and Denmark). As cannabis use was only measured among 15 year olds and two countries (Norway and Greece) did not assess cannabis use, this resulted in *N* = 56,159 in 32 countries for the analyses on cannabis use.

### Measures

*Substance use* Across all waves, substance use was assessed as follows. *Weekly alcohol use.* Students were asked how often they drank beer, wine, and liquor/spirits. For each type, response options were “1 = never”, ”2 = rarely”, “3 = every month”, “4 = every week”, “5 = every day”. This variable was dichotomized by combining options 1 to 3 (indicating less than weekly alcohol use, coded as 0) and 4 to 5 (to reflect at least weekly drinking, coded as 1). *Weekly smoking.* Smoking status was defined based on the question “How often do you smoke tobacco at present?” Original answer categories (never, less than weekly, weekly but not daily, daily) were recoded into *weekly smoking* (1) and *less than weekly smoking* (0). *Lifetime cannabis use.* Students reported the frequency of cannabis use in their lifetime on a scale from 1 to 7, with 1 = never and 7 = 40 times or more. Answers were recoded into 0 = *never* and 1 = *at least once*.

*Electronic media communication (EMC)* In 2002, 2006 and 2010, electronic media communication was measured by the item “How often do you talk to your friend(s) on the phone or send them text messages or have contact through the internet?” Response options were “never or rarely”, “1 or 2 days a week”, “3 or 4 days a week”, “5 or 6 days a week”, and “every day”. Answers were recoded into 0 = less than daily and 1 = daily. In 2014, electronic media communication was measured by means of three items: How often do you a) contact your friends using texting/SMS?; b) actively contact your friends using instant messaging (e.g. BBM, Facebook chat)?; c) contact your friends using other social media, such as Facebook (posting on wall, not chat), My Space, Twitter, apps (e.g. Instagram), games (e.g. Xbox), YouTube? Answer categories were “hardly ever or never”, “less than weekly”, “weekly”, “daily”. Answers were recoded into 0 = less than daily and 1 = daily.

*Face*-*to*-*face (FTF) contact with peers in the evening* was measured by the item “How many evenings per week do you usually spend out with your friends after 8 PM?” in 2002, 2006 and 2010. The response options ranged from zero to seven evenings a week. Answers were recoded into 0 = less than daily and 1 = daily. In 2014, the question was changed into “How often do you meet your friends outside school time after 8 PM?” Answer categories were hardly ever or never, less than weekly, weekly, and daily. As prevalence rates in the first categories were relatively low, we combined them into the category “less than once a week”.

*Confounders* In our analyses, we controlled for age, gender and family affluence. Family affluence was measured by the ridit-based relative HBSC Family Affluence Scale (Torsheim et al. 2016).

### Statistical analyses

Analyses were performed on two distinct datasets, as EMC was assessed differently in 2014, compared to the other years (2002, 2006, 2010). Using the trend data (2002–2006–2010), we assessed descriptive statistics of trends over time in adolescent EMC, FTF, and substance use (Table 1). Correlations were calculated between two (absolute) difference scores (2002–2006 and 2002–2010) of EMC, FTF and adolescent substance use across countries. Next, hierarchical multilevel analyses (with the second-level variable being country) were performed to test the association of daily EMC with three indicators of substance use (tobacco, alcohol and cannabis; Table 2). Analyses were controlled for age (tobacco and alcohol), gender, and family affluence (tobacco). In Model 1, survey year was added to the model, followed by daily EMC (Model 2), daily FTF (Model 3), and the interaction of survey year × EMC (Model 4).

**Table 1:** Adolescent face-to-face contact with peers in the evening, electronic media communication and substance use in 26 countries, 2002–2010 (%)

Region	Country	Daily face-to-face contact with peers in the evening		Daily electronic media communication		Weekly alcohol use		Weekly tobacco use		Lifetime cannabis use	
		2002	Change 02–10	2002	Change 02–10	2002	Change 02–10	2002	Change 02–10	2002	Change 02–10
Western Europe											
	Austria	5.1	+ 0.8	23.5	+ 23.1	9.8	+ 2.0	12.0	+ 0.1	13.7	− 0.1
	Belgium	8.3	− 2.0	25.1	+ 18.7	12.8	− 4.7	9.9	− 3.2	25.4	− 4.9
	France	8.0	− 1.2	11.8	+ 26.7	7.3	− 1.1	11.3	− 2.7	30.0	− 3.2
	Germany	12.9	− 3.4	26.8	+ 6.0	12.7	− 6.4	15.5	− 9.4	23.9	− 12.9
	Netherlands	15.8	− 9.5	19.5	+ 11.5	13.3	− 6.5	10.1	− 3.3	25.9	− 5.4
	Switzerland	7.1	− 2.3	27.2	+ 9.0	10.4	− 1.8	11.0	− 3.3	44.7	− 15.4
Northern Europe											
	Denmark	12.5	− 4.0	34.6	+ 21.8	17.1	− 8.8	7.5	− 1.9	23.4	− 8.3
	Estonia	25.2	− 6.8	23.8	+ 17.8	9.6	− 3.7	12.3	− 3.0	17.2	+ 4.6
	Latvia	20.4	+ 2.8	18.8	+ 25.6	7.9	− 0.9	12.0	+ 0.2	11.8	+ 13.5
	Lithuania	17.3	− 1.8	13.8	+ 48.2	10.0	− 2.8	12.4	− 0.3	7.9	+ 13.4
	Finland	33.4	− 2.8	30.8	+ 13.0	5.1	− 1.5	13.6	− 5.4	10.3	− 0.9
	Sweden	14.2	− 1.8	26.9	+ 26.4	6.8	− 4.4	6.6	− 1.1	6.8	–
	UK	33.7	− 10.2	33.7	+ 15.9	22.3	− 12.2	10.8	− 4.8	37.6	− 17.4
Eastern Europe											
	Czech Rep.	16.4	− 2.6	23.5	+ 16.8	19.2	+ 0.8	14.2	− 1.8	30.5	+ 0.0
	Estonia	25.2	− 6.8	23.8	+ 17.8	9.6	− 3.7	12.3	− 3.0	17.2	+ 4.6
	Poland	13.3	+ 1.6	19.5	+ 28.3	7.4	− 1.4	11.2	− 3.7	18.1	+ 0.7
	Russia	35.6	− 1.8	46.4	+ 5.3	14.3	− 8.3	12.3	− 2.7	13.6	− 5.2
	Ukraine	28.0	+ 2.1	24.9	+ 19.4	18.7	− 2.6	16.7	− 7.6	23.4	− 12.4
	Macedonia	13.9	+ 4.3	36.2	+ 9.7	6.8	+ 0.6	5.7	− 0.2	3.1	− 0.3
Southern Europe											
	Croatia	13.2	+ 2.9	42.7	+ 5.5	13.3	+ 3.4	9.7	+ 2.7	16.2	− 2.8
	Greece	9.0	+ 0.2	38.2	+ 7.2	16.1	− 2.0	6.2	+ 0.2	5.2	+ 1.8
	Italy	10.7	− 1.7	34.7	+ 14.2	23.0	− 10.9	10.0	− 0.8	21.4	− 1.8
	Portugal	5.0	− 2.4	25.9	+ 29.2	8.0	− 4.2	11.3	− 6.1	19.8	− 8.5
	Slovenia	12.1	− 2.0	33.6	+ 8.2	10.8	+ 0.4	10.3	− 2.7	28.4	− 5.2
Non-European											
	Canada	24.1	− 3.4	40.5	+ 7.0	10.6	− 4.9	6.8	− 1.3	44.3	− 10.4
	Israel	20.2	− 8.4	42.3	+ 11.2	15.3	− 1.9	7.9	− 1.4	5.9	− 0.5

–, No data available

**Table 2:** Results of multilevel logistic regression analyses predicting adolescent substance use in 26 countries, 2002–2010, odds ratios

	Weekly alcohol use				Weekly tobacco use				Lifetime cannabis use^a^			
	Model 1	Model 2	Model 3	Model 4	Model 1	Model 2	Model 3	Model 4	Model 1	Model 2	Model 3	Model 4
Survey year												
2002	Ref.	Ref.	Ref.		Ref.	Ref.	Ref.		Ref.	Ref.	Ref.	
2006	0.76** [0.75–0.78]	0.71** [0.70–0.73]	0.72** [0.70–0.74]		0.74** [0.72–0.76]	0.67** [0.65–0.69]	0.68** [0.66–0.70]		0.81** [0.78–0.83]	0.75** [0.72–0.77]	0.76** [0.74–0.79]	
2010	0.58** [0.57–0.60]	0.52** [0.51–0.54]	0.54** [0.53–0.56]		0.71** [0.70–0.73]	0.63** [0.61–0.64]	0.66** [0.64–0.68]		0.75** [0.72–0.78]	0.66** [0.64–0.69]	0.70** [0.67–0.72]	
Daily electronic media communication		1.92** [1.88–1.96]	1.71** [1.67–1.75]			2.04** [1.99–2.08]	1.69** [1.64–1.73]			1.81** [1.75–1.86]	1.59** [1.54–1.64]	
Daily face-to-face contact with peers in the evening			2.34** [2.28–2.40]				3.90** [3.80–4.00]				2.80** [2.70–2.89]	
Survey year × daily electronic media communication												
2002				Ref.				Ref.				Ref.
2006				0.88** [0.83–0.92]				0.98 [0.93–1.04]				0.89* [0.83–0.96]
2010				0.93* [0.88–0.98]				1.05 [0.99–1.11]				1.01 [0.94–1.09]

Analyses are controlled for age (tobacco, alcohol), gender (all substances), and family affluence (all substances)

**p < 0.001; *p < 0.01

^a^The analysis on cannabis use was based on 25 countries, as Swedish trend data on cannabis use were not available

We also provide descriptive statistics of adolescent EMC, FTF, and substance use, by country, in 2014 (Table 3). Also here, hierarchical multilevel logistic regression models (with the second-level variable being country) were applied (Table 4). Analyses were controlled for age (tobacco and alcohol), gender, and family affluence (tobacco). In Model 1, daily EMC was added to the model, followed by FTF (Model 2), and the country-level mean prevalence of daily EMC (Model 3). Lastly, we added a cross-level interaction between daily EMC and the aggregated (average) EMC use per country (Model 4). Here, we divided countries into tertiles based on the percentage of youth that engaged in EMC at a daily basis. All analyses were performed using Stata SE 12.1 (College Station, Texas, USA).

**Table 3:** Adolescent face-to-face contact with peers in the evening, electronic media communication and substance use in 34 countries in 2014, %

Region	Country	Daily face-to-face contact with peers in the evening	Daily electronic media communication	Weekly alcohol use	Weekly tobacco use	Lifetime cannabis use
Western Europe						
	Austria	5.2	83.5	8.7	6.6	9.5
	Belgium	3.7	64.4	6.6	5.0	17.7
	France	5.0	76.6	6.6	7.8	28.2
	Germany	4.4	64.6	6.5	6.2	16.7
	Luxembourg	12.4	64.8	7.1	8.0	18.0
	Netherlands	5.3	63.5	5.9	4.4	15.9
	Switzerland	2.9	88.1	4.4	4.5	23.7
Northern Europe						
	Denmark	5.2	63.4	6.2	4.1	15.7
	Estonia	4.8	58.0	4.5	5.4	24.3
	Latvia	6.2	50.9	3.6	5.8	20.5
	Finland	7.9	82.7	4.8	5.8	9.6
	Norway	11.9	53.6	1.5	1.8	–
	Sweden	5.3	63.9	2.7	2.9	5.8
	UK	8.7	68.5	6.0	3.6	17.9
Eastern Europe						
	Albania	15.8	51.3	11.6	2.8	5.5
	Armenia	14.5	50.7	10.2	1.8	1.5
	Bulgaria	33.7	72.1	16.3	11.5	22.7
	Czech Rep.	3.6	38.7	9.6	6.3	23.1
	Hungary	13.1	57.0	11.6	7.9	12.9
	Macedonia	22.0	70.8	7.2	4.1	3.2
	Rep. of Moldova	8.5	27.6	12.4	3.6	5.7
	Poland	6.9	54.5	6.4	8.0	23.6
	Romania	17.5	41.0	13.7	9.6	7.5
	Russia	14.2	64.2	6.0	8.6	8.6
	Ukraine	11.3	48.3	13.7	5.7	7.2
Southern Europe						
	Croatia	12.5	64.9	14.1	9.5	15.2
	Greece	14.0	54.2	11.6	5.7	–
	Italy	7.6	80.4	13.4	8.6	22.0
	Malta	4.8	53.1	11.1	4.4	13.3
	Portugal	2.2	33.6	4.4	4.2	11.7
	Slovenia	2.9	47.4	8.6	5.1	21.1
Non-European						
	Canada	11.2	59.4	5.7	3.3	25.5
	Israel	9.2	63.9	13.2	6.7	6.5

–, No data available

**Table 4:** Results of multilevel logistic regression analyses predicting adolescent substance use in 34 countries in 2014, odds ratios

	Weekly alcohol use					Weekly tobacco use				Lifetime cannabis use^a^			
	Model 1		Model 2	Model 3	Model 4	Model 1	Model 2	Model 3	Model 4	Model 1	Model 2	Model 3	Model 4
Daily electronic media communication	1.70** [1.47–1.96]	1.34** [1.15–1.57]		1.34** [1.15–1.57]		1.89** [1.59–2.24]	1.33* [1.10–1.62]	1.33* [1.10–1.62]		1.83** [1.61–2.07]	1.33** [1.15–1.54]	1.32** [1.14–1.53]	
Face-to-face contact with peers in the evening													
Rarely or < once a week		Ref.		Ref.			Ref.	Ref.			Ref.	Ref.	
Weekly		2.17** [2.07–2.28]		2.17** [2.07–2.28]			2.59** [2.45–2.74]	2.59** [2.45–2.74]			2.54** [2.40–2.69]	2.54** [2.40–2.69]	
Daily		3.35** [3.17–3.54]		3.35** [3.17–3.54]			5.37** [5.02–5.73]	5.36** [5.02–5.73]			4.25** [3.94–4.59]	4.25** [3.94–4.59]	
Country-level % of youth engaged in daily electronic media communication													
Low				Ref.				Ref.				Ref.	
Medium				0.98 [0.58–1.66]				1.34 [0.87–2.05]				1.50 [0.91–2.47]	
High				1.10 [0.65–1.86]				1.58† [1.02–2.45]				1.59 [0.97–2.59]	
Daily electronic media communication × country-level % of youth engaged in daily electronic media communication													
Low					Ref.				Ref.				Ref.
Medium					1.27 [0.88–1.83]				1.10 [0.69–1.73]				1.05 [0.74–1.50]
High					1.26 [0.87–1.83]				1.30 [0.82–2.07]				1.03 [0.73–1.46]

Analyses are controlled for age (tobacco, alcohol), gender (all substances), and family affluence (tobacco)

**p < 0.001; *p < 0.01; ^†^*p* < 0.05

^a^The analysis on cannabis use was based on 32 countries, as Norwegian and Greek data on cannabis use were not available in 2014

## Results

### Can the increase in EMC explain the decline in substance use between 2002 and 2010?

Descriptive statistics of adolescent EMC, FTF and substance use in the period 2002–2010 are presented in Table 1. Substance use decreased in virtually all countries and regions between 2002 and 2010. Some Eastern European countries (Croatia, Estonia, Latvia, Lithuania) show increasing trends for at least one substance of at least 1%. FTF declined in most countries, except for Croatia, Latvia, Poland, Ukraine and Macedonia. EMC increased considerably in all countries, with increases ranging from 5.3% (Russia) to 48.2% (Lithuania). Figure 1 in the Online appendix provides a graphical presentation of the trends per country.

When correlating the change in EMC with the change in adolescent substance use across countries across time, we did not find significant associations (see supplementary table for an overview of all correlation coefficients). While the association was significant for cannabis use between 2002 and 2010 (*r* = 0.47, *p* = 0.02), we repeated the analysis leaving out Latvia and Lithuania, as they show remarkably deviant trends regarding cannabis use (i.e. a strong increase, while 18 out of 25 countries showed a decrease). This resulted in a non-significant finding (*r* = 0.12, *p* = .60). Thus, even though EMC increased and substance use decreased in many countries between 2002 and 2010, the trends over time appear to be distinct and unrelated.

We also correlated country-level changes in FTF with country-level changes in substance use and EMC. Trends in EMC were not related to trends in FTF. However, positive links were found between changes in FTF and changes in substance use, especially between 2002 and 2006 (smoking: *r* = .41, *p* = 0.04; alcohol: *r* = .63, *p* = 0.0005; cannabis: *r* = .42, *p* = 0.04). Between 2002 and 2010, the trends were only associated for alcohol use (*r* = .58, *p* = 0.002).

### EMC, FTF and substance use: different associations across time (2002–2006–2010)?

Multilevel logistic regression models indicated that substance use overall declined between 2002 and 2006 and even more so between 2002 and 2010 (Table 2). Adolescents engaging in daily EMC and daily FTF were more likely to report substance use. Interestingly, adding EMC to the trend analysis strengthened rather than weakened the time trends in substance use. It can thus be concluded that *in spite of* (rather than because of) the increase in EMC, substance use decreased between 2002 and 2010. Adding FTF to the model slightly weakened the trends in adolescent substance use and substantially reduced the association between EMC and substance use. Finally, the interaction between time and EMC was not significant for smoking, but it was significant for alcohol and cannabis use. A post hoc plot showed that the positive associations between EMC and substance use were stronger in 2006 (alcohol and cannabis) and 2010 (alcohol), as compared to 2002.

### EMC, FTF and substance use in 2014: different associations across countries?

Descriptive statistics of EMC, FTF, and substance use in 2014 are presented in Table 3. EMC and FTF differed considerably across countries, with EMC ranging from 27.6% in the Republic of Moldova to 88.1% in Switzerland and FTF ranging from 2.2% (Portugal) to 33.7% (Bulgaria). Weekly alcohol use ranged from 1.9% (Iceland) to 16.3% (Bulgaria). Weekly tobacco use ranged from 1.5% (Iceland) to 11.5% (Bulgaria) and lifetime cannabis use among 15 year olds ranged from 1.5% (Armenia) to 28.2% (Belgium).

Hierarchical multilevel logistic regression models were applied to test the association of EMC with substance use across countries while controlling for age, gender, and family affluence (Table 4). Overall, EMC was positively associated with the three types of substance use (Model 1, Table 4). FTF was also positively associated with all three substances (Model 2, Table 4). Adding FTF to the model reduced the strength of the associations between EMC and substance use, but all associations remained significant. Thus, FTF partly explained the positive association between EMC and substance use.

The next step was to examine if the association between EMC and substance use differed across countries, and if so, if this cross-national variation could be explained by the popularity of EMC among youth in a given country. We first examined whether there was sufficient variability between countries to justify a random slope for EMC. The difference in log likelihood between a model with a random and a fixed slope was significant (*p*s < 0.01), indicating that associations between EMC and substance use differed across countries. To test whether the cross-national variation in these associations could be explained by the country-level popularity of EMC among youth, we added the country-level mean prevalence of daily EMC and its interaction with individual EMC as predictors to the model (Model 3 and 4, respectively). The interactions between individual daily EMC and the average country-level EMC were not significant, indicating that the cross-national variability in the association between EMC and substance use cannot be explained by the country-level popularity of EMC among adolescents.

To gain more insight into the cross-national differences in the association between EMC and substance use, we examined these associations in each country separately (not in a table). In the majority of countries, associations were positive, but in a few countries, there was a negative association between EMC and alcohol use (Russian Federation, Israel, Iceland) and tobacco use (Israel).

### Supplementary analyses

As age and gender relate strongly to adolescent substance use and EMC, we examined whether the associations found in this study differed across age groups and between boys and girls. Interaction terms (gender × EMC; age × EMC) were added to Model 1 of Table 4. The association of EMC with smoking was stronger for girls than for boys (OR = 1.26, *p* < 0.001), but no gender differences were found in the associations with alcohol and cannabis. For both smoking and alcohol, associations with EMC were stronger for older adolescents (ORs up to 1.76, *p*s < 0.001). For cannabis, no interaction with age was performed as it was only measured among 15 year olds.

## Discussion

This study provides no support for the hypothesis that recent declines in adolescent substance use, reported in the majority of western countries, can be explained by increasing EMC among adolescents during the same period. First, on a national level, declines in substance use over time were *not* associated with increases in EMC, while they were associated with declines in FTF. Second, across countries and time, adolescents who reported daily EMC spent *more* time with friends in the evenings and were *more* likely to use substances, than adolescents who did not report daily EMC. Although positive associations between EMC and substance use were found in the vast majority of countries, the associations varied between countries. This cross-national variation could not be explained by the country-level use of EMC among youth. Finally, for alcohol and cannabis use, the positive association with EMC varied in strength across time, with stronger associations in more recent survey years.

Overall, our findings provide support for the stimulation rather than the displacement hypothesis, suggesting that EMC overall functions as a social connector for adolescents. It seems to intensify already existing friendships, rather than replace face-to-face peer interactions. As adolescents often engage in substance use in the presence of peers (Branstetter et al. 2011; Chassin et al. 2009), adolescents who tend to use EMC are may be more likely to report substance use.

In line with our hypotheses, the positive links between EMC and substance use were stronger in more recent years, although this was primarily the case for alcohol (2006 and 2010) and cannabis use (2006), as compared to 2002. The idea behind this hypothesis was that increased use of EMC enhances the function of EMC as a social connector which, in turn, affects the association with substance use. There are several reasons why these results were particularly revealed for alcohol use. First, we have reason to believe that alcohol use is typically consumed for social reasons by young people (Kuntsche et al. 2005), making associations between EMC and alcohol use more likely in contexts in which EMC serves more as a social connector. Second, the increased popularity of EMC over time has led to an increase in online advertisements, especially for alcohol (Anderson et al. 2009; Nicholls 2012; Pennay et al. 2015), leading to an increased association of EMC to alcohol use. Third, many adolescents use EMC to display engagement in substance use, and the most frequently displayed substance is alcohol (e.g. texting about or posting pictures of partying and drinking; Loss et al. 2013). The more prevalent EMC became, the more adolescents may have been exposed to such displays, which may have strengthened the association between EMC and alcohol use.

In contrast to our hypotheses, the link between EMC and substance use did not vary with the prevalence of EMC in a particular country. Potentially, social norms regarding substance use in different countries have a larger effect on this link than the prevalence of EMC. For example, the association between EMC and substance use may depend on the extent to which substance use is part of popular youth culture. In countries with relatively low prevalence rates of substance use (such as Iceland), the association with EMC may be different as substance use is not integrated in popular youth culture, while EMC is. Moreover, the extent to which parents supervise the EMC of their children, or the extent to which it is culturally accepted that adolescents spend (unsupervised) time together at night with friends, may differ across countries. Such factors are also likely to affect links between EMC, FTF, and substance use.

Although we predominantly found positive associations between EMC and substance use across countries and time, our study does not rule out that EMC is related to *less* FTF and *less* substance use for specific groups of adolescents. For example, socially anxious and lonely adolescents seem to prefer EMC over FTF communication (Pierce 2009) and more strongly value the opportunity to communicate anonymously online (Valkenburg and Peter 2007b, 2011). This suggests that for socially anxious and lonely adolescents, EMC does not necessarily lead to new friendships, more FTF or more substance use. Future research is needed to gain more insight into these individual differences in the potential effects of EMC on adolescent substance use.

Our findings suggest that, rather than an increase in EMC, a decrease in FTF may have played a role in the declining trends in adolescent substance use between 2002 and 2010. Among others, the decrease in FTF in the evening might be the consequence of increasingly strict parental rule-setting and monitoring, which has been reported in some European countries (e.g. De Looze et al. 2017) and in the USA (Twenge 2017). If parents are less likely to allow their children to go out at night, they restrict their children’s exposure to contexts in which substances are typically used. Potentially, the rise of the Internet technology in a broader sense has enabled parents to monitor their children more easily, for instance because it has become more common for children to have their own cell phone. The increase in substance use prevention programs across countries (Kuntsche and Ravens-Sieberer 2015) and stricter rules regarding the use and purchase of substance use across Europe (e.g. ban of smoking in public areas, ban of selling tobacco and alcohol to minors) may also have encouraged parents to become stricter regarding their child’s exposure to contexts that could lead to substance use. An alternative explanation for the decline in FTF in the evenings may be that youth are becoming more involved in structured and constructive activities during the day. While evidence from the USA does not seem to support this hypothesis (Twenge 2017), European trend data on leisure time use are scarce.

### Strengths and limitations

A major strength of this study lies in the use of large and nationally representative samples of adolescents in a sizeable number of countries which are heterogeneous in terms of, for example, their sociocultural and policy backgrounds. Moreover, our study compromises a time span of 12 years, which provided us with the unique opportunity to correlate national-level changes in EMC, FTF and substance use over time. The inclusion of data from different countries and different moments in time increases the external validity of our study. However, this study should be interpreted with knowledge of its limitations. First, due to the repeated cross-sectional design, no causal inferences can be made. Adolescents who are at risk of substance use may also be at risk of higher levels of EMC (Osaki et al. 2012), or adolescents who use substances use may be more drawn to EMC because their substance use enhances their social connectedness and standing among peers (Killeya-Jones et al. 2007). Second, although we included an important set of social and individual factors in our models and showed that they contributed to substance use independently, a more elaborate model of substance use should also include biological, genetic and personality factors, as well as their interactions. Such a model provides opportunities for examining whether EMC use is differently associated with substance use for specific groups of adolescents (e.g. excessive users; active versus passive users; socially anxious adolescents; (e.g. Blinka et al. 2015; Boniel-Nissim et al. 2015b). Third, while adolescents are fairly accurate in their self-reported substance use (e.g. Harrison et al. 2007), the self-reported nature of all measures in this study may have led to biased estimates.

## Conclusion

Not the increase in EMC, but rather the decrease in FTF may have played a role in the decline in adolescent substance use in the early twenty-first century. This study suggests that adolescents who engage in daily EMC interact with friends relatively often, online as well as offline, and are more likely to engage in substance use. We call for future studies to replicate our findings and examine links between EMC, FTF and substance use in a longitudinal design.

## Electronic supplementary material

Below is the link to the electronic supplementary material.

Supplementary material 1 (DOCX 3751 kb)

## References

[CR1] Anderson P, de Bruijn A, Angus K, Gordon R, Hastings G (2009). Impact of alcohol advertising and media exposure on adolescent alcohol use: a systematic review of longitudinal studies. Alcohol Alcohol.

[CR2] Blinka L, Škařupová K, Ševčíková A, Wölfling K, Müller KW, Dreier M (2015). Excessive internet use in European adolescents: What determines differences in severity?. Int J Public Health.

[CR3] Boniel-Nissim M, Lenzi M, Zsizos E, Gaspar de Matos M, Gommans R, Harel-Fisch Y, Djalovski A, Van der Sluijs W (2015). International trends in electronic media communication (EMC) among 11- to 15-year-olds (2002–2010): Does EMC affect ease of communication with friends from the opposite sex?. Eur J Public Health.

[CR4] Boniel-Nissim M, Tabak I, Mazur J, Borraccino A, Brooks F, Gommans R, van der Sluijs W, Zsiros E, Craig W, Harel-Fisch Y, Finne E (2015). Supportive communication with parents moderates the negative effects of electronic media use on life satisfaction during adolescence. Int J Public Health.

[CR5] Branstetter SA, Low S, Furman W (2011). The influence of parents and friends on adolescent substance use: multidimensional approach. J Subst Use.

[CR6] Bryant JA, Sanders-Jackson A, Smallwood AMK (2006). IMing, text messaging, and adolescent social networks. J Comput Mediat Commun.

[CR7] Chassin L, Hussong A, Barrera M, Molina BSG, Trim R, Ritter J, Lerner R, Steinberg L (2009). Adolescent substance use. Handbook of adolescent psychology.

[CR8] Currie C, Roberts C, Morgan A, Smith R, Settertobulte W, Samdal O, Barnekow V (eds.) (2004) Young people's health in context: International report from the HBSC 2001/02 survey, (Health Policy for Children and Adolescents, No.4). WHO Regional Office for Europe, Copenhagen.

[CR10] Currie C, Inchely J, Molcho M et al (eds) Health Behavior in School-aged Children (HBSC) study protocol: background, methodology and mandatory items for the 2013/14 survey St Andrews, Scotland: Child and Adolescent Health Research Unit, University of St Andrews, 2014 (http://www.hbscorg/news/indexaspx?ni=2418. Accessed 1 April 2018

[CR11] De Looze M, Raaijmakers Q, ter Bogt T, Bendsten P, Farhat T, Ferreira M, Godeau E, Kuntsche E, Molcho M, Pförtner TK, Simons-Morton B, Vollebergh W, Pickett W (2015). Decreases in weekly alcohol use in Europe and North America: evidence from 28 countries from 2002 to 2010. Eur J Public Health.

[CR12] De Looze M, van Dorsselaer S, Monshouwer K, Vollebergh W (2017). Trends in adolescent alcohol use in the Netherlands, 1992–2015. Differences across sociodemographic groups and links with strict parental rule-setting. Int J Drug Policy.

[CR14] Eurostat (2018) Internet access and use statistics – households and individuals. Available at http://ec.europa.eu/eurostat/statistics-explained/index.php/Internet_access_and_use_statistics_-_households_and_individuals

[CR15] Gommans R, Stevens GWJM, Finne E (2015). Frequent electronic media communication with friends is associated with higher adolescent substance use. Int J Public Health.

[CR16] Haddon L, Livingstone S, Online Kids, EU Kids Online network (2012). EU Kids Online: national perspectives, EU Kids Online.

[CR17] Harrison LD, Martin SS, Enev T, Harrington D (2007) Comparing drug testing and self-report of drug use among youths and young adults in the general population (DHHS Publication No. SMA 07-4249, Methodology Series M-7). Rockville, MD: Substance Abuse and Mental Health Services Administration, Office of Applied Studies

[CR18] Hublet A, de Looze M, Fotiou A, Donnelly P, Vilhjalmsson R, Baska T, Aasvee K, Franelic IP, Nic Gabhainn S, ter Bogt T (2015). Trends in the co-occurrence of tobacco and cannabis use in 15-year-olds from 2002 to 2010 in 28 countries of Europe and North America. Eur J Public Health.

[CR19] Inchley J, Currie D, Young T (2016). Growing up unequal: gender and socioeconomic differences in young people’s health and well-being Health Behaviour in School-aged Children (HBSC) study: international report from the 2013/2014 survey.

[CR20] Killeya-Jones LA, Nakajima R, Costanzo PR (2007). Peer standing and substance use in early-adolescent grade-level networks: a short-term longitudinal study. Prev Sci.

[CR21] Kraut R, Patterson M, Lundmark V (1998). Internet paradox: a social technology that reduces social involvement and psychological well-being?. Am Psychol.

[CR22] Kraut R, Kiesler S, Boneva B (2002). Internet paradox revisited. J Soc Issues.

[CR23] Kuntsche E, Ravens-Sieberer U (2015). Monitoring adolescent health behaviours and social determinants cross-nationally over more than a decade: introducing the Health Behaviour in School-aged Children (HBSC) study supplement on trends. Eur J Public Health.

[CR24] Kuntsche E, Knibbe R, Gmel G, Engels R (2005). Why do young people drink? A review of drinking motives. Clin Psychol Rev.

[CR25] Kuntsche E, Simons-Morton B, ter Bogt T (2009). Electronic media communication with friends from 2006 to 2010 and links to face-to-face contacts in adolescence: an HBSC study in 31 European and North American countries and regions. Int J Public Health.

[CR26] Livingston M (2014). Trends in non-drinking among Australian adolescents. Addiction.

[CR27] Loss J, Lindacher V, Curbach J (2013). Do social networking sites enhance the attractiveness of risky health behaviour? Impression management in adolescents’ communication on Facebook and its ethical implications. Public Health Issues.

[CR29] Mesch GS (2003). The family and the internet: the Israeli case. Soc Sci Q.

[CR30] Nicholls J (2012). Everyday, everywhere: alcohol marketing and social media—current trends. Alcohol Alcohol.

[CR31] Nie NH (2001). Sociability, interpersonal relationships and the internet: reconciling conflicting findings. Am Behav Sci.

[CR32] Nie NH, Hillygus DS, Erbring L, Wellman B, Haythornthwaite C (2002). Internet use, interpersonal relations, and sociability: a time diary study. The internet in everyday life.

[CR33] Osaki Y, Ohida T, Kanda H, Kaneita Y, Kishimoto T (2012). Mobile phone use does not discourage adolescent smoking in Japan. Asian Pac J Cancer Prev.

[CR34] Pennay A, Livingston M, MacLean S (2015). Young people are drinking less: it is time to find out why. Drug Alcohol Rev.

[CR35] Pierce T (2009). Social anxiety and technology: face-to-face communication versus technological communication among teens. Comput Hum Behav.

[CR36] Subrahmanyam K, Greenfield P (2008). Online communication and adolescent relationships. Future Child.

[CR37] Torsheim T, Cavallo F, Levin KA (2016). Psychometric validation of the revised family affluence scale: a latent variable approach. Child Indic Res.

[CR38] Twenge JM (2017). Why today’s super-connected kids are growing up less rebellious, more tolerant, less happy—and completely unprepared for adulthood and what that means for the rest of us.

[CR39] Valkenburg PM, Peter J (2007). Online communication and adolescent well-being: testing the stimulation versus the displacement hypothesis. J Comput Mediat Commun.

[CR40] Valkenburg PM, Peter J (2007). Preadolescents’ and adolescents’ online communication and their closeness to friends. Dev Psychol.

[CR41] Valkenburg PM, Peter J (2011). Online communication among adolescents: an integrated model of its attraction, opportunities, and risks. J Adol Health.

[CR42] Weiser EB (2001). The functions of Internet use and their psychological consequences. Cyberpsychol Behav.

