# Supplementary material for: The decline in adolescent substance use across Europe and North America in the early twenty-first century: A result of the digital revolution?
Source: Int J Public Health. 2018 Dec 17;64(2):229–40. doi: 10.1007/s00038-018-1182-7 (PMC6439251; doi:10.1007/s00038-018-1182-7)
Supplement: Supplementary file 1 — Supplementary material 1 (DOCX 3751 kb) [file 38_2018_1182_MOESM1_ESM.docx]

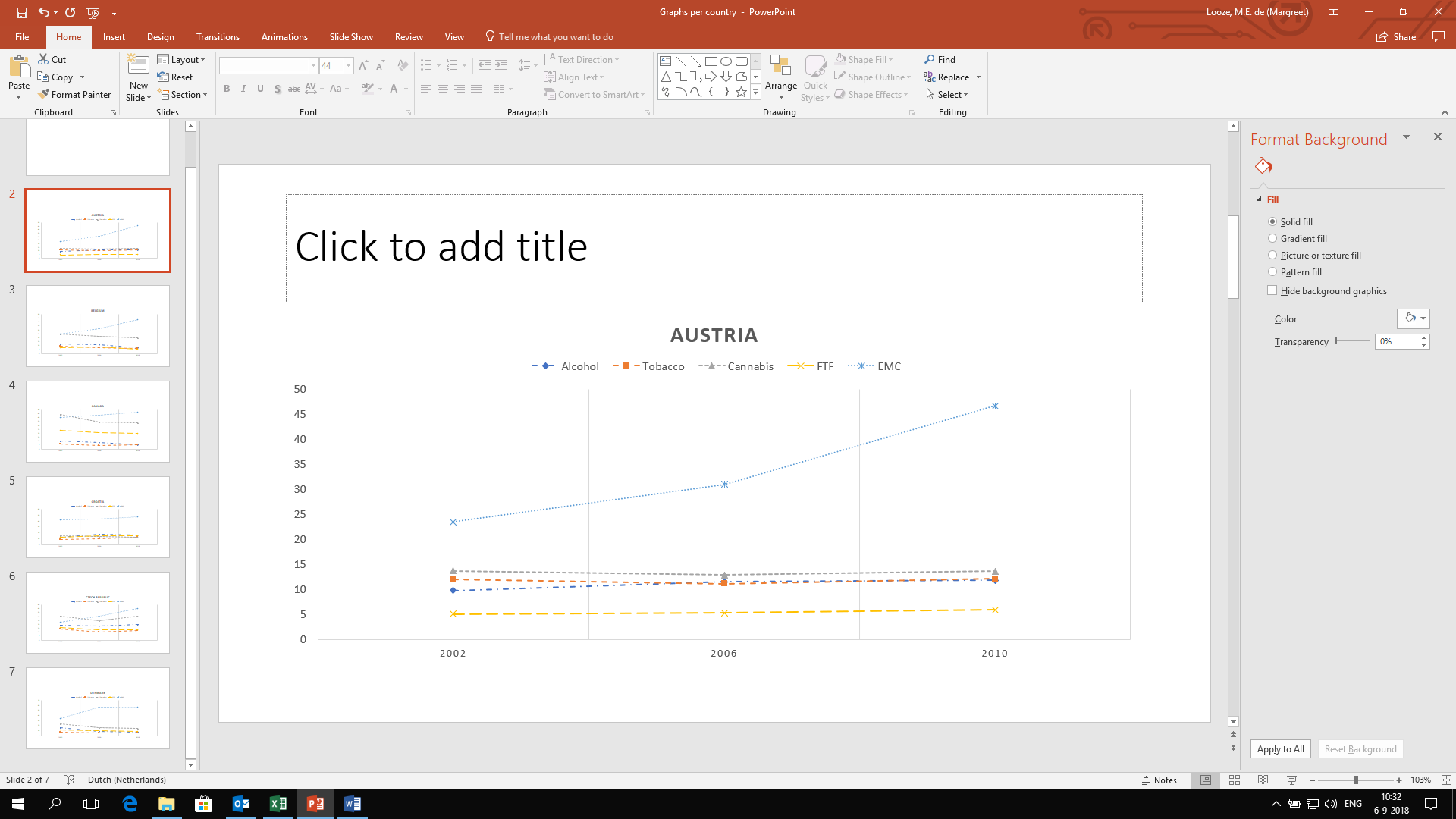

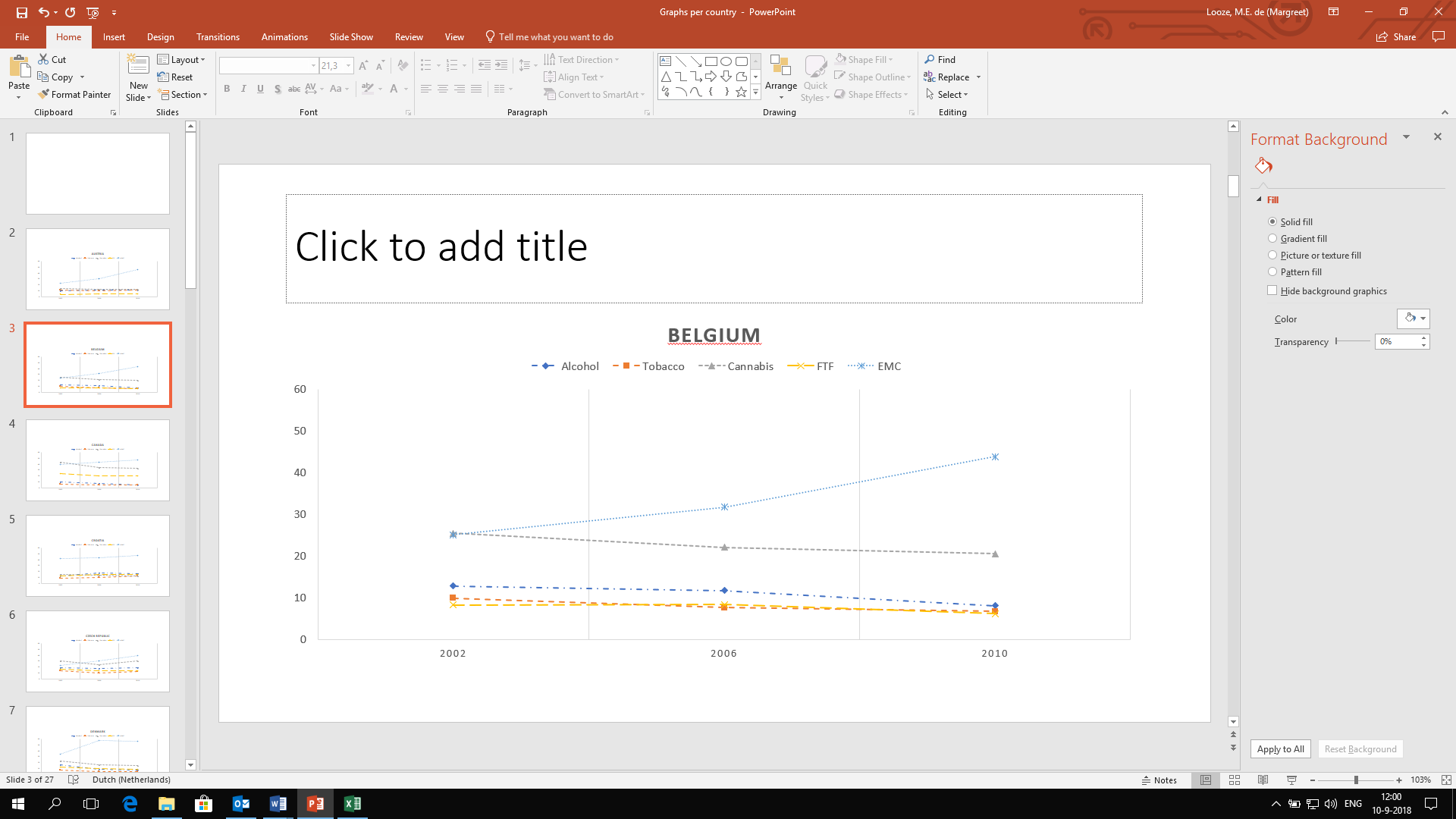


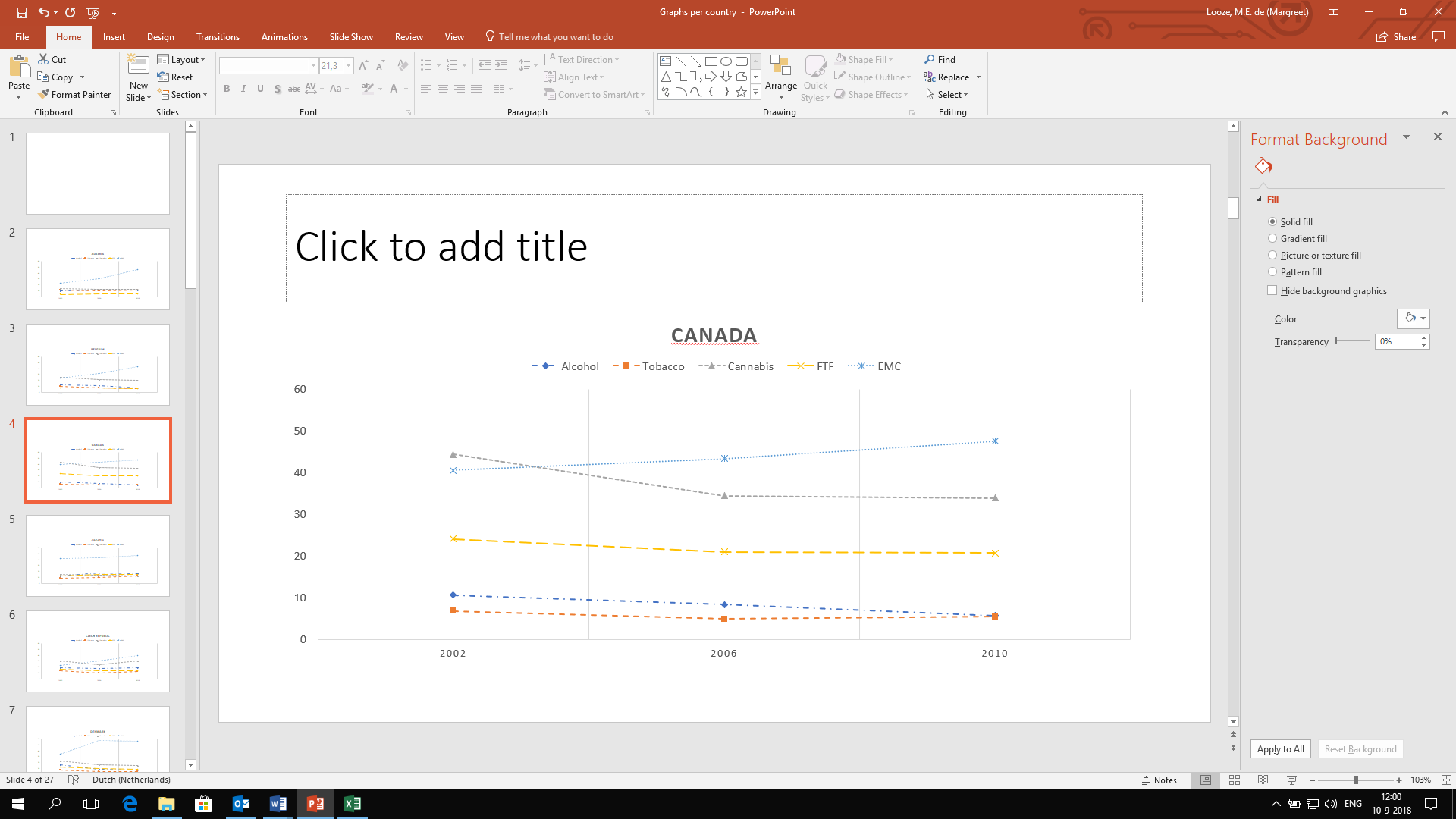

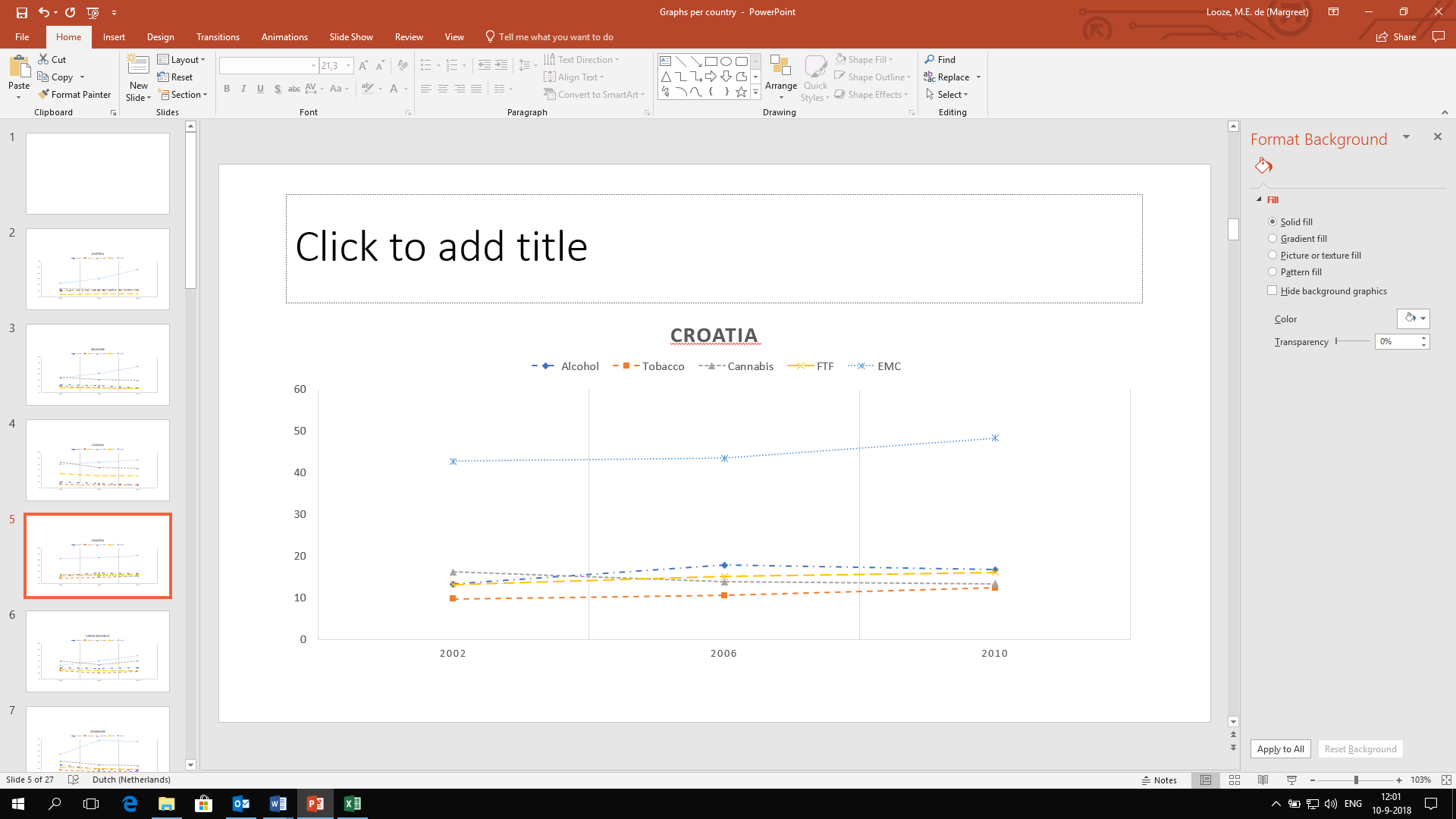


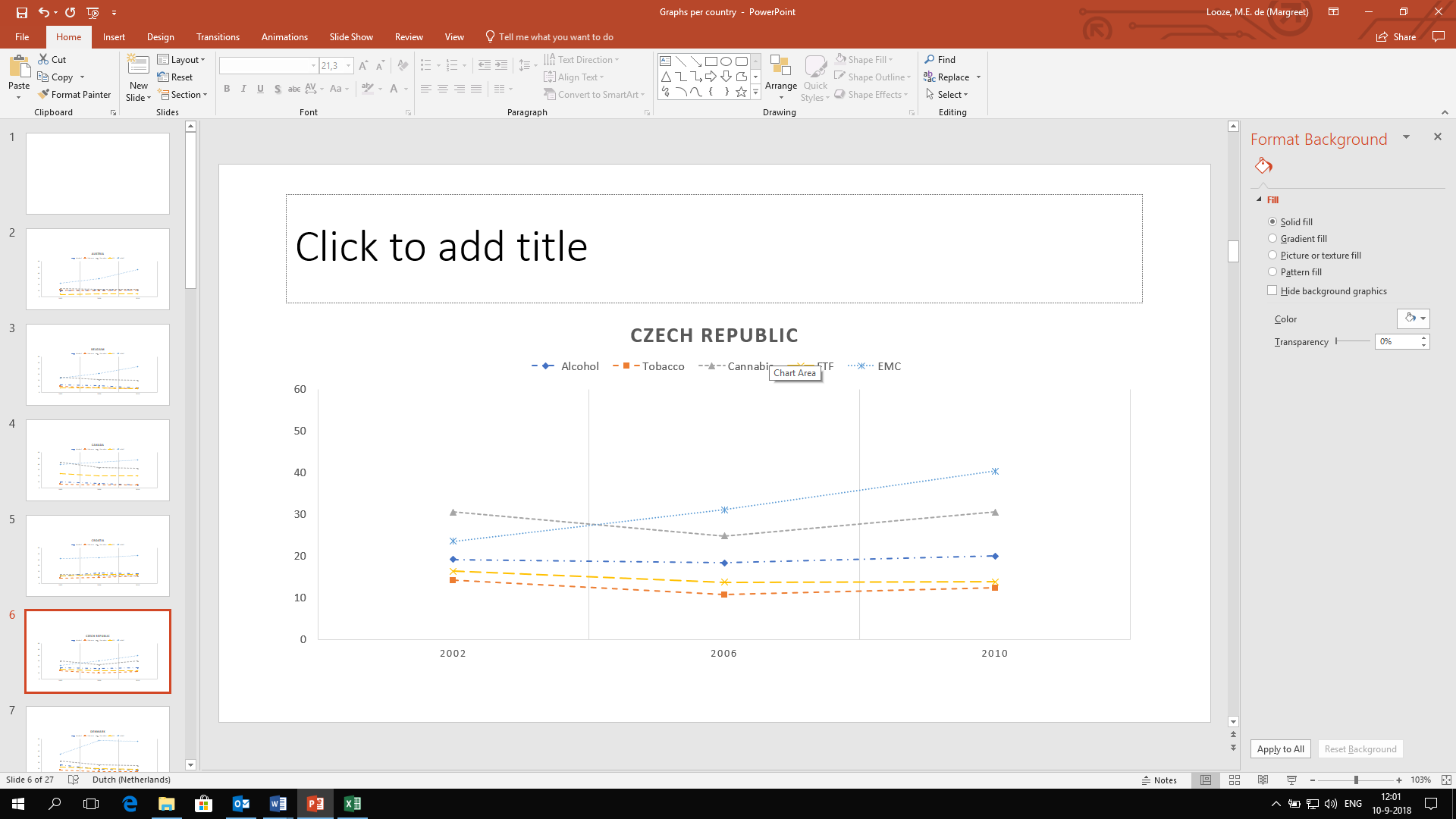

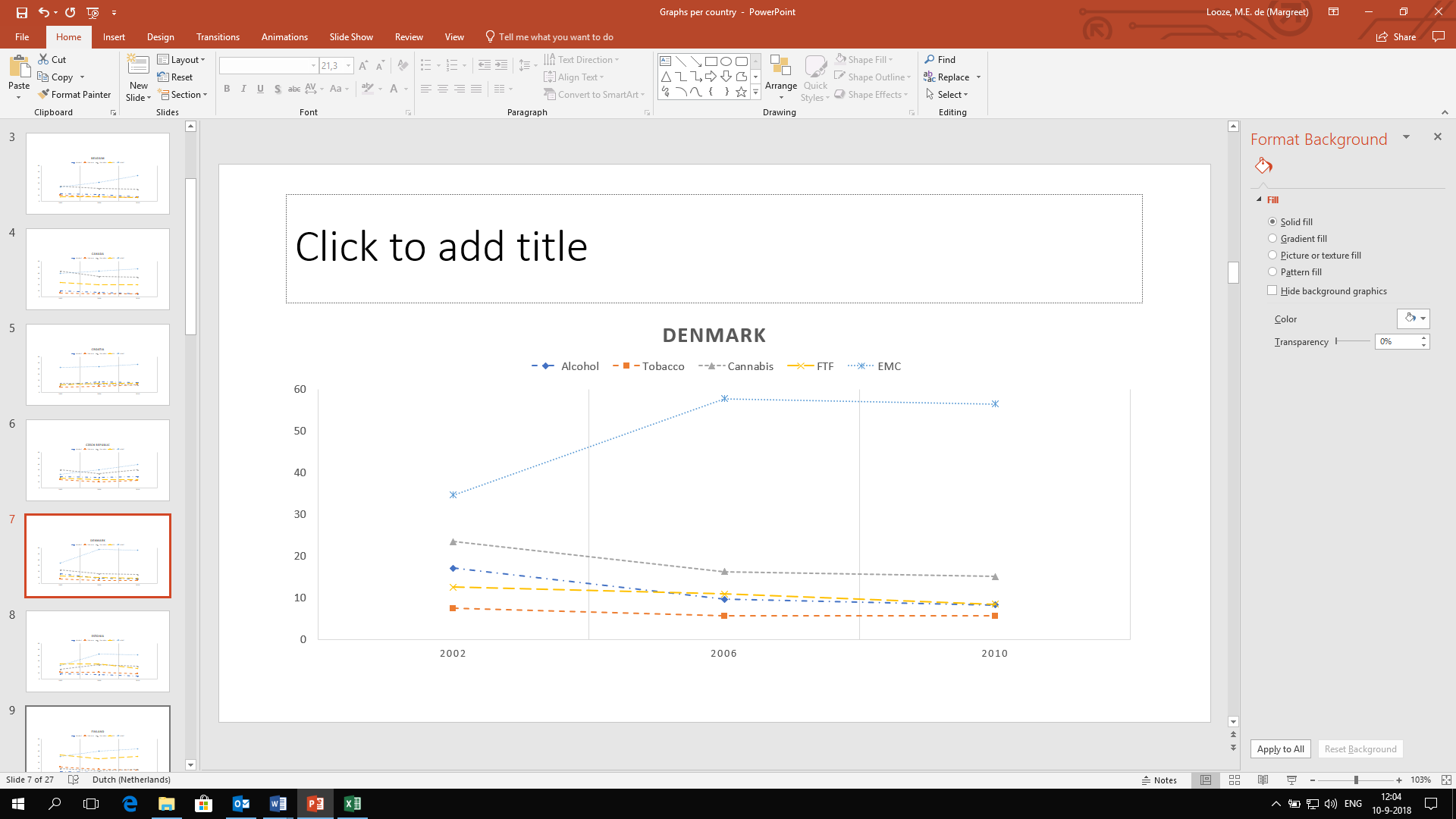


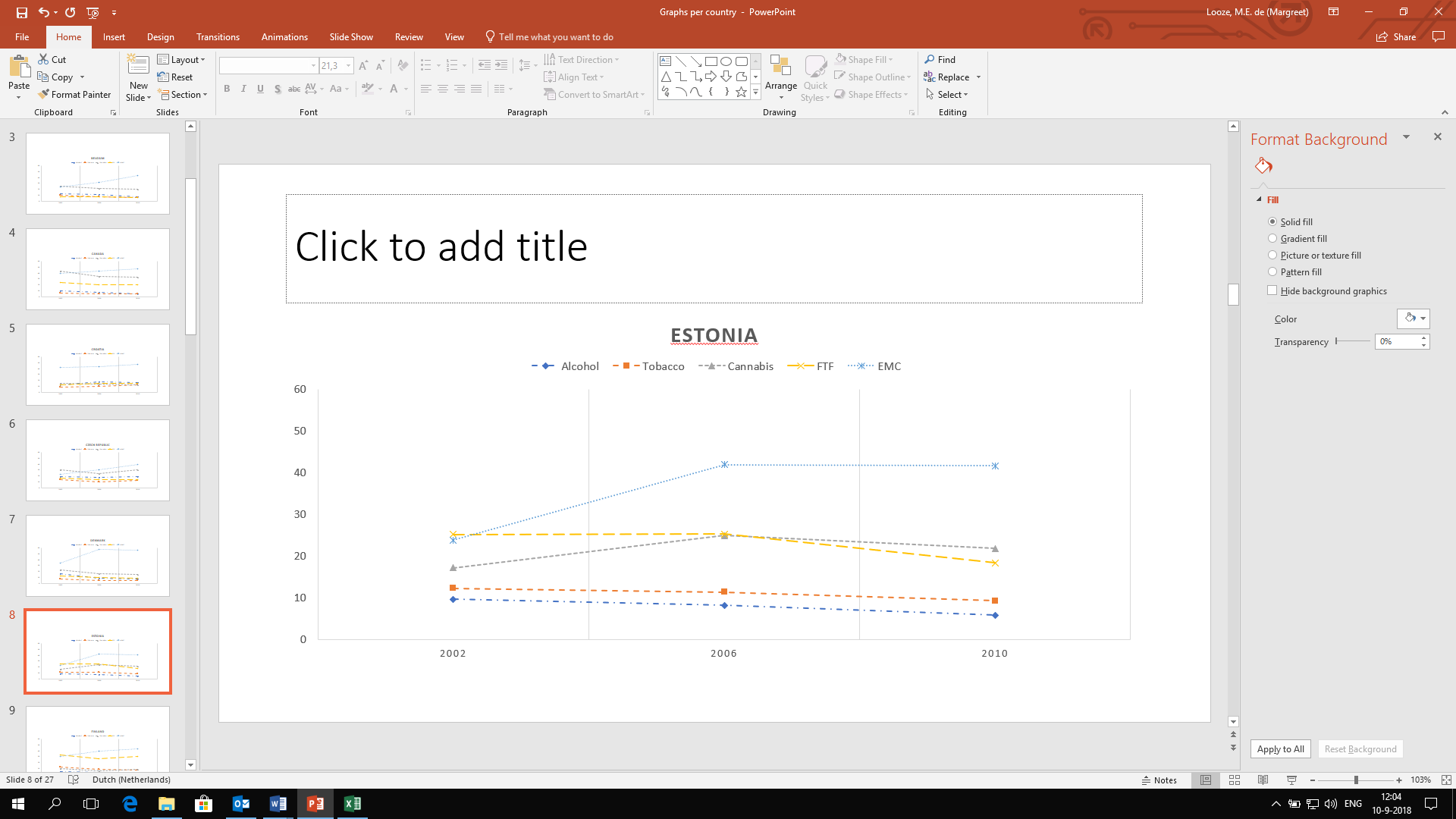

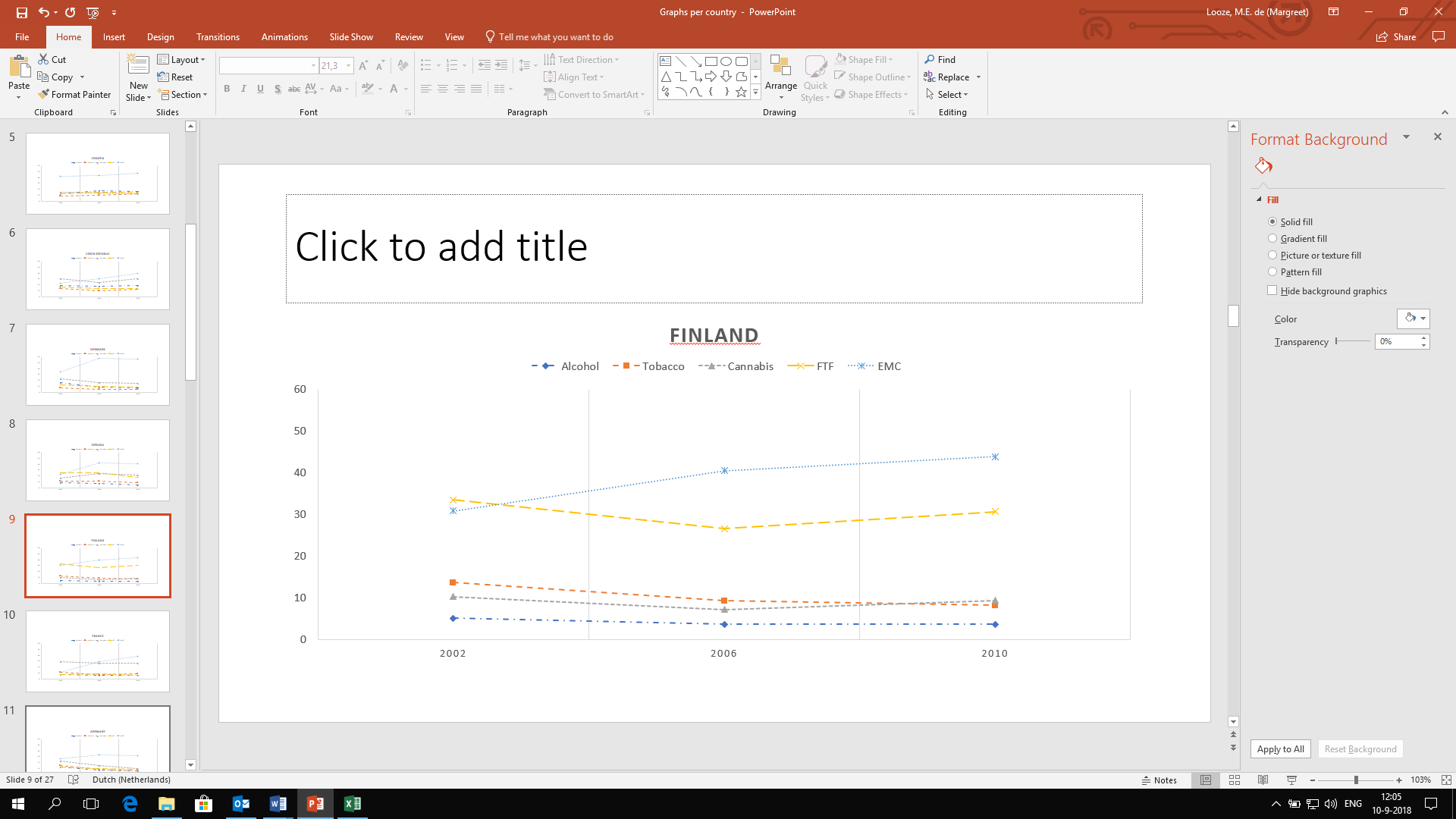


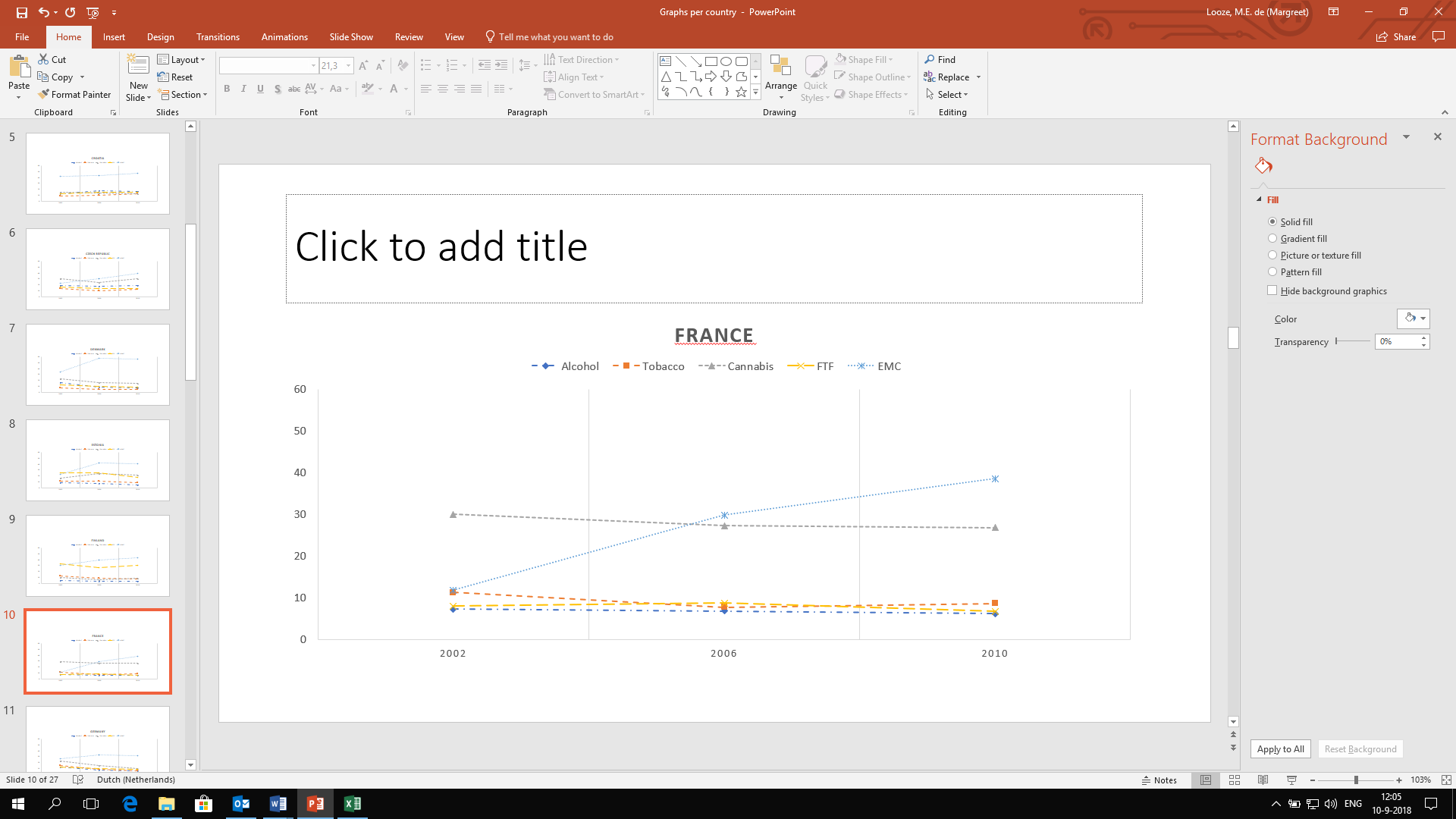

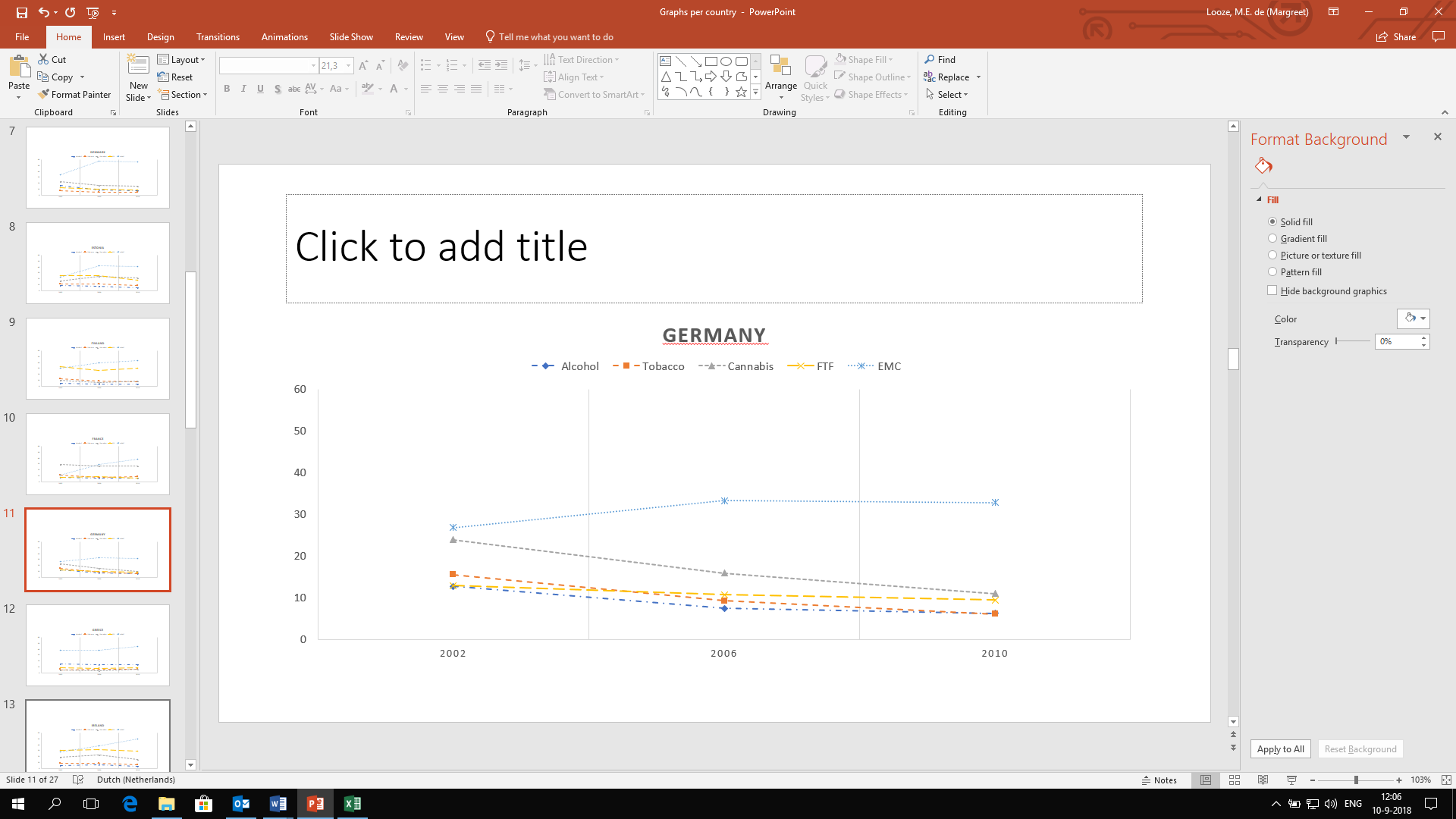


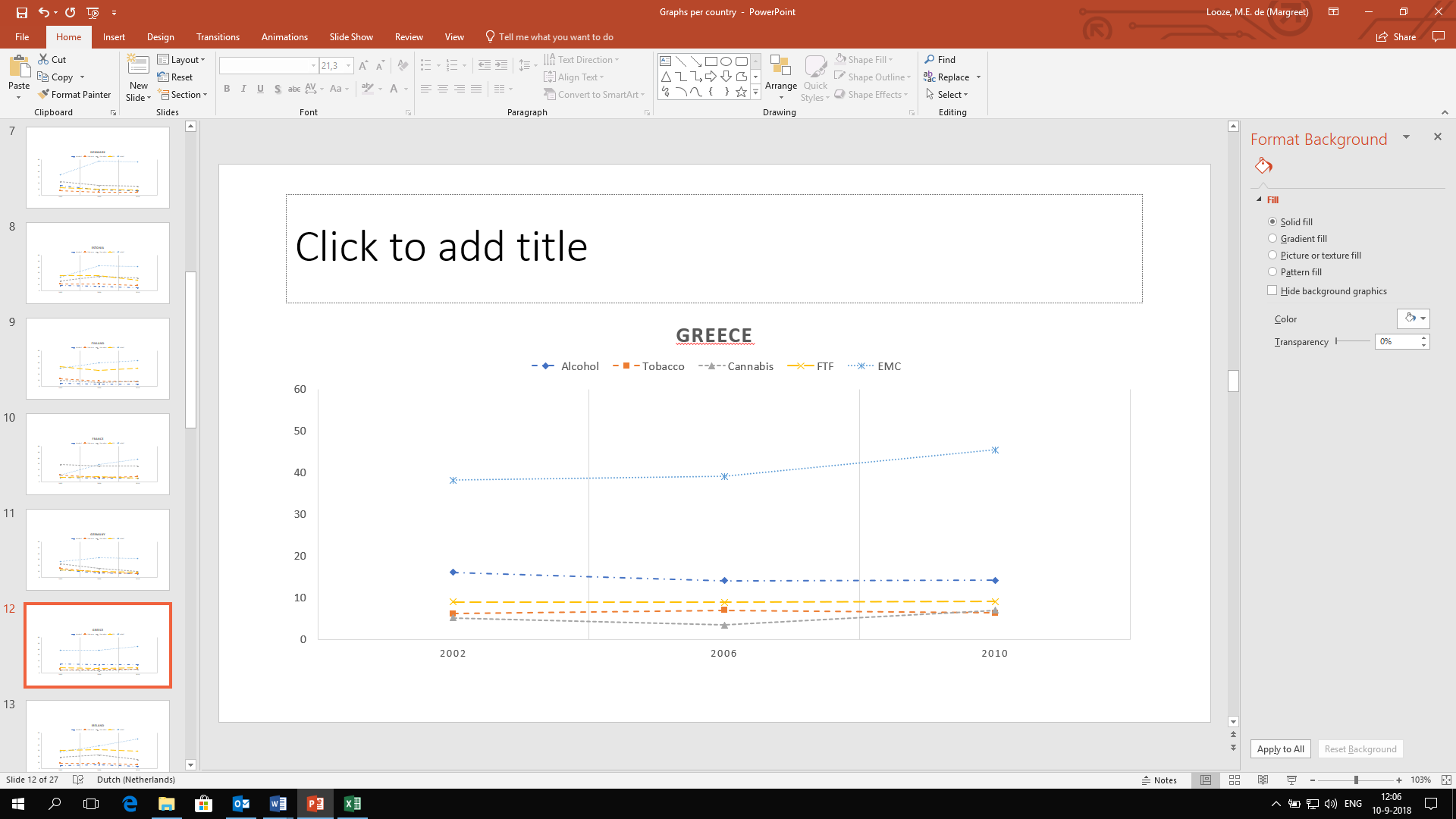

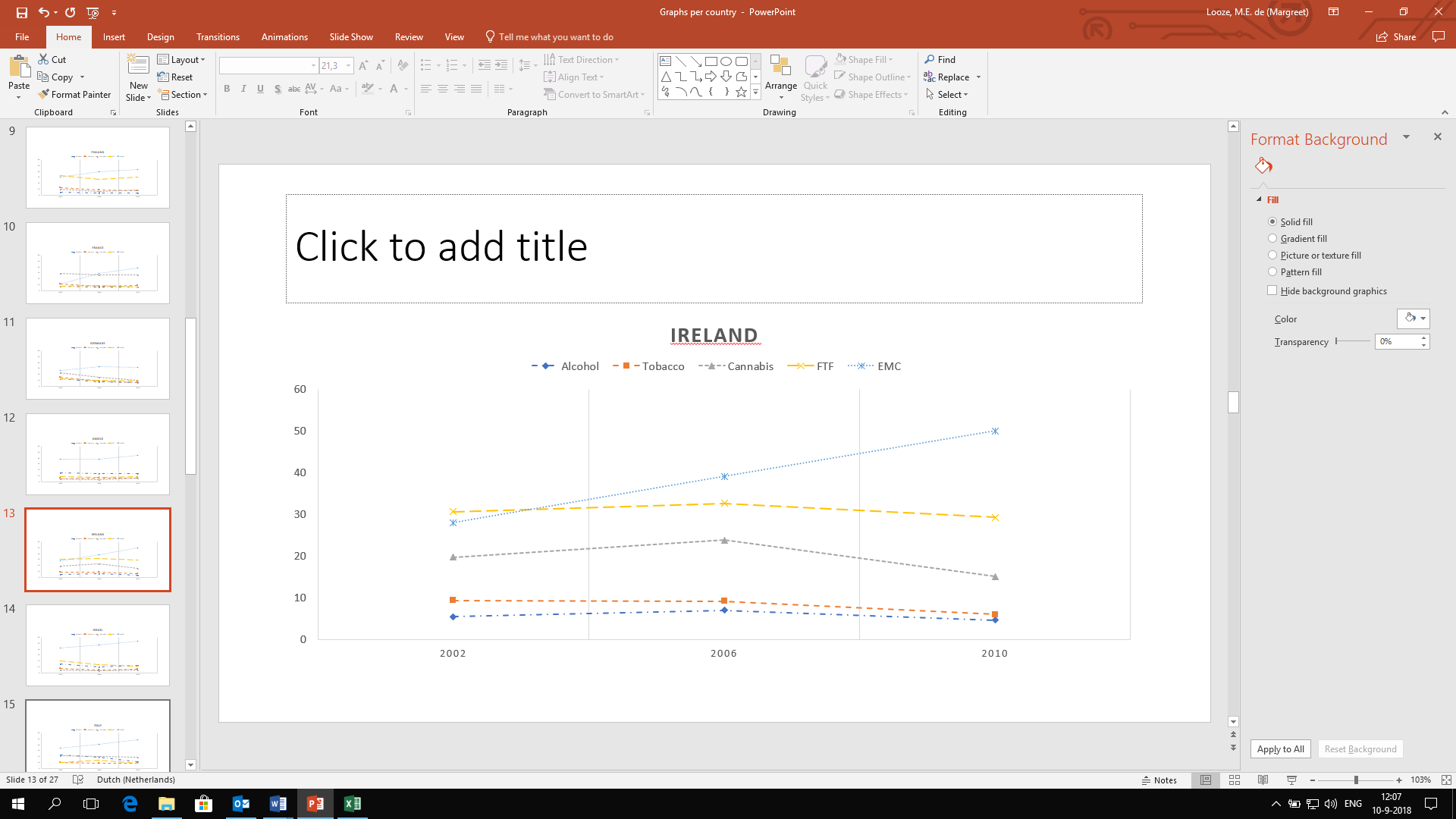


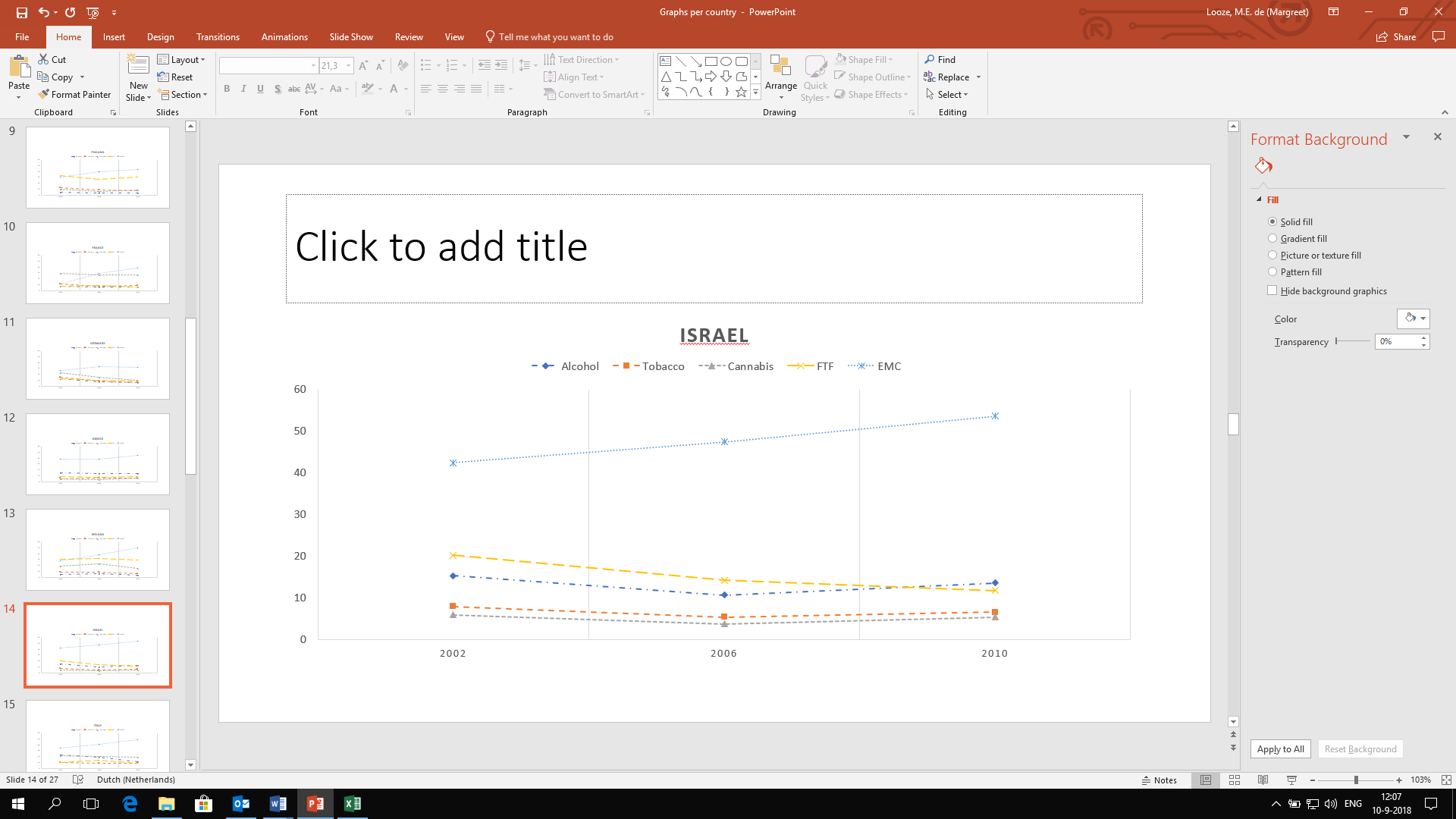

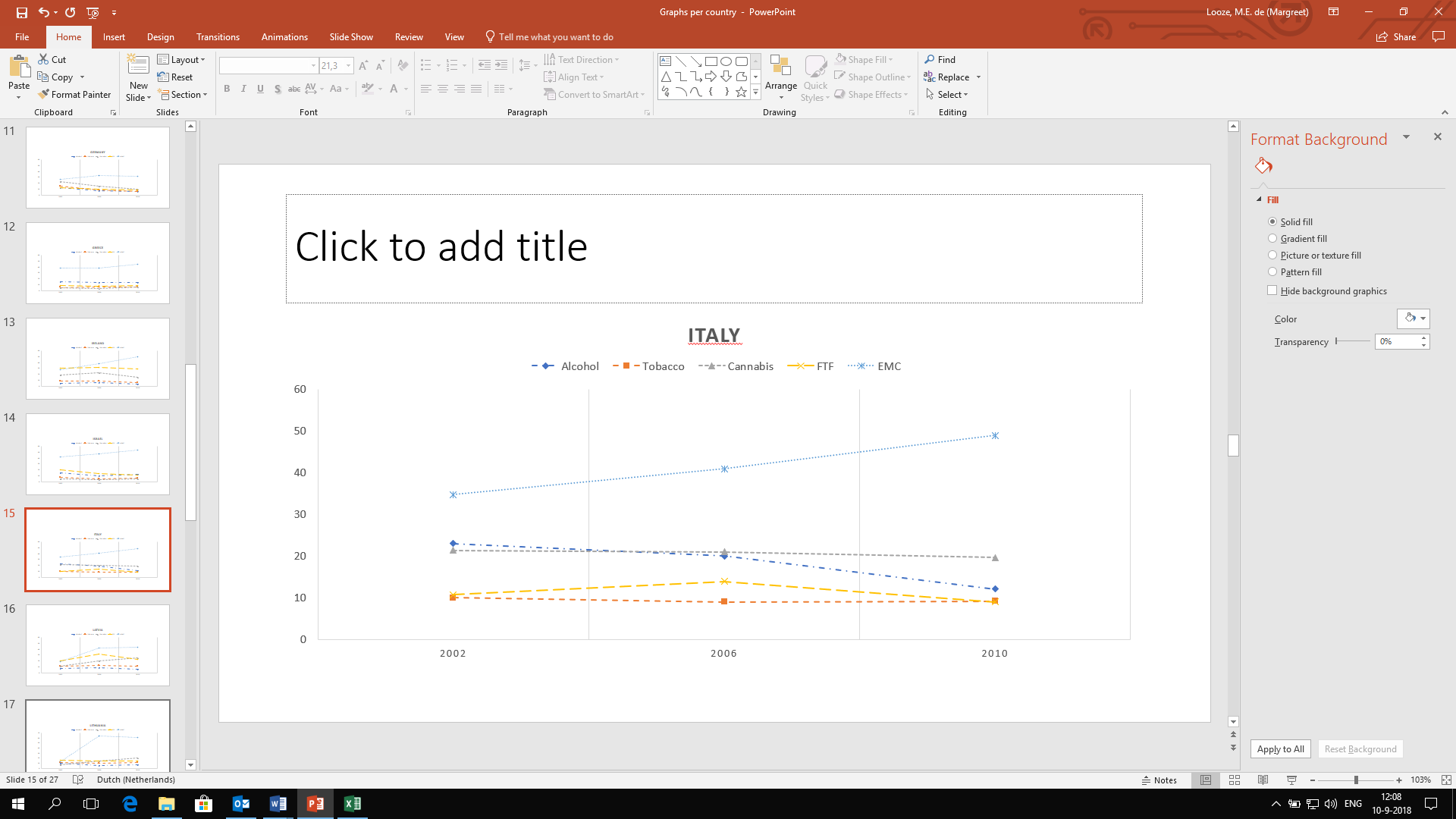


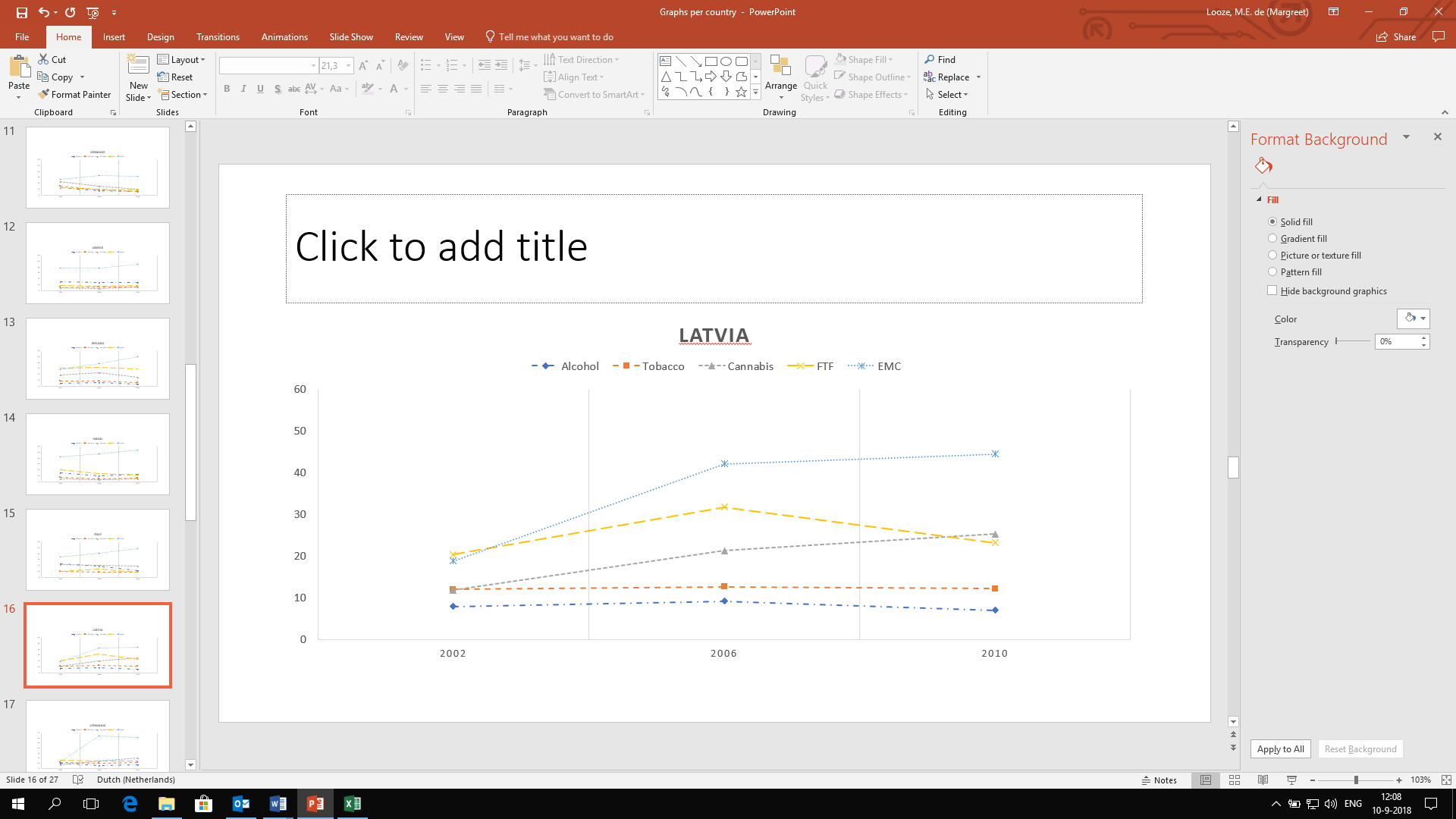

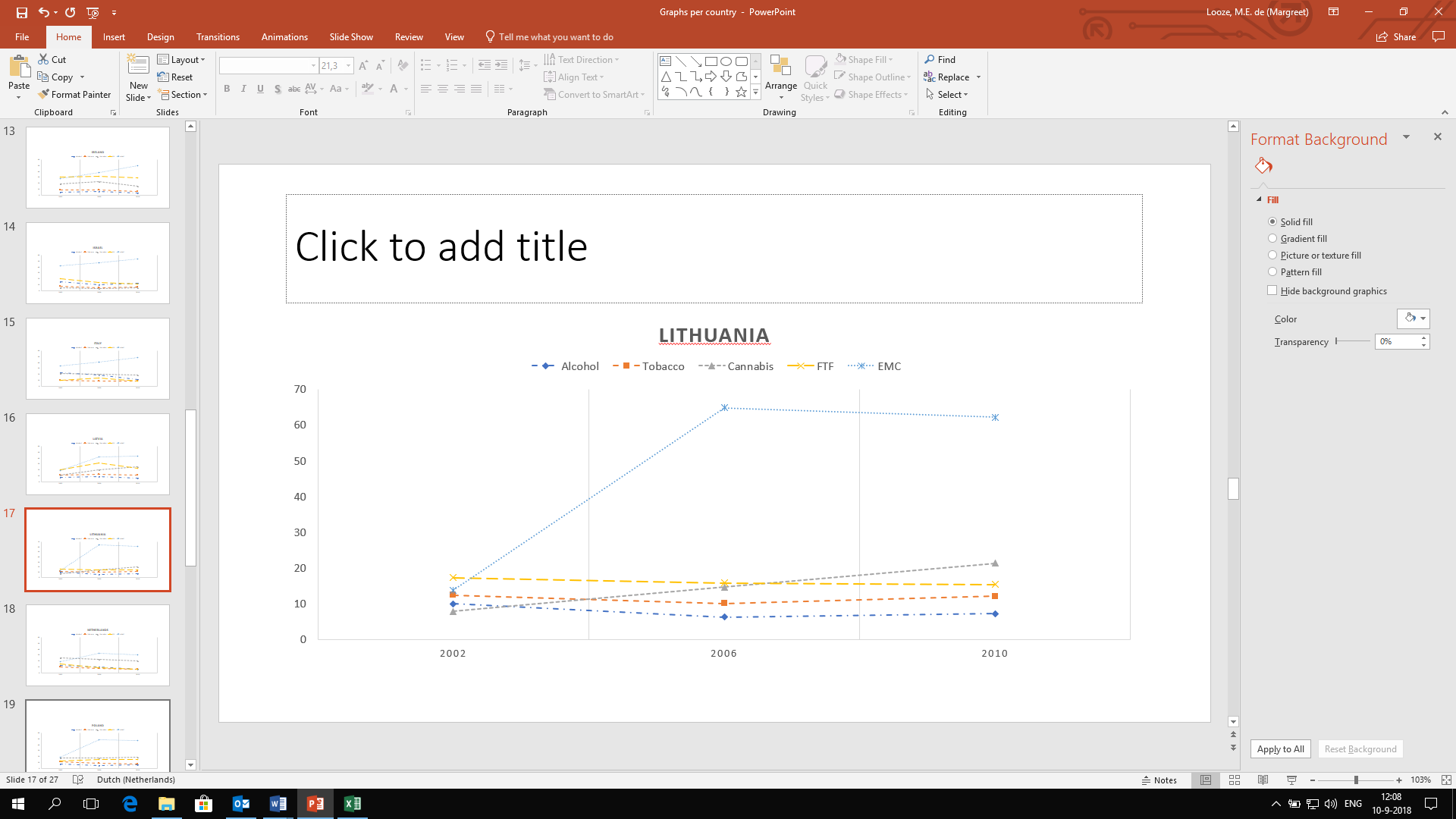


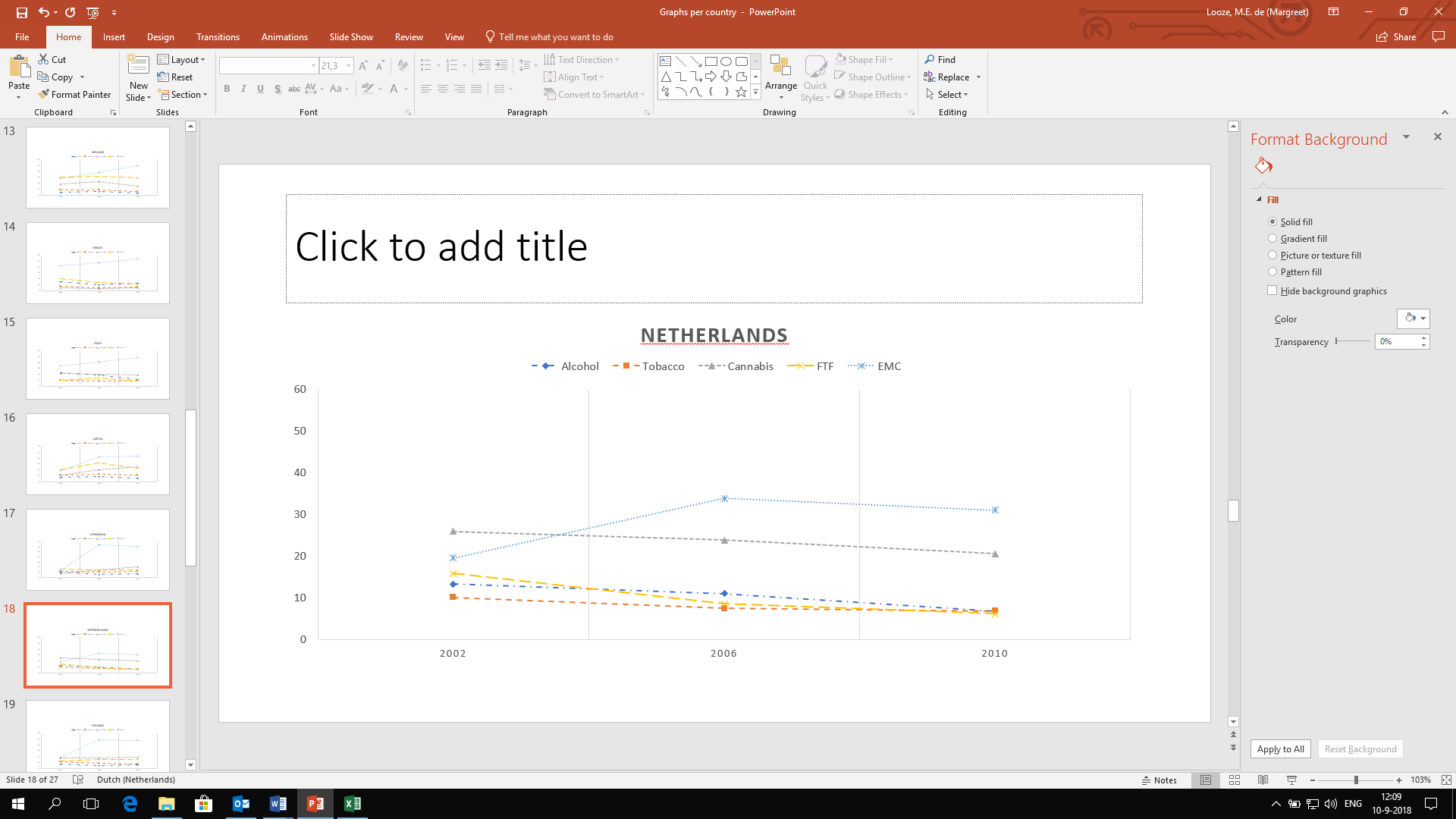

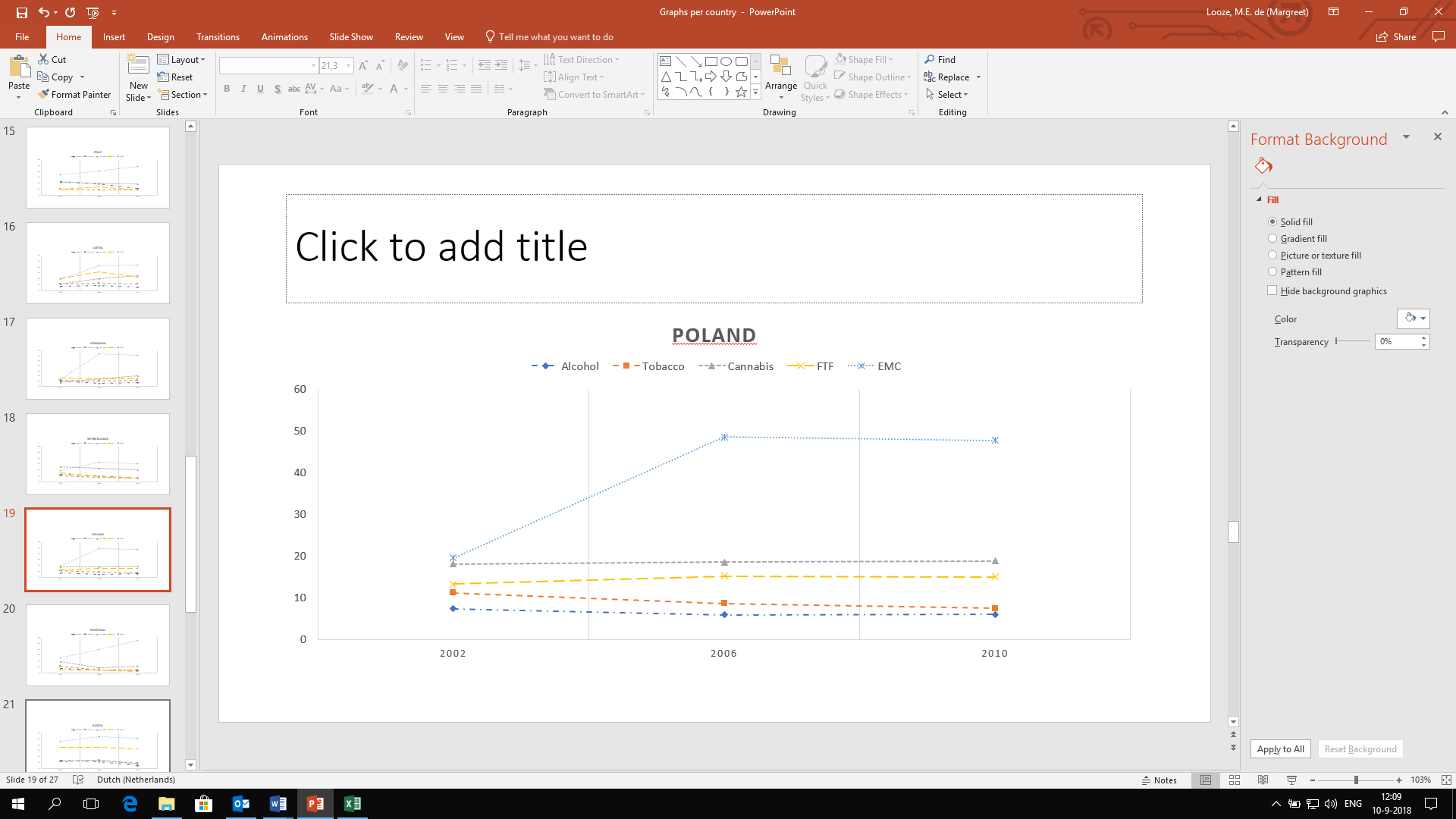


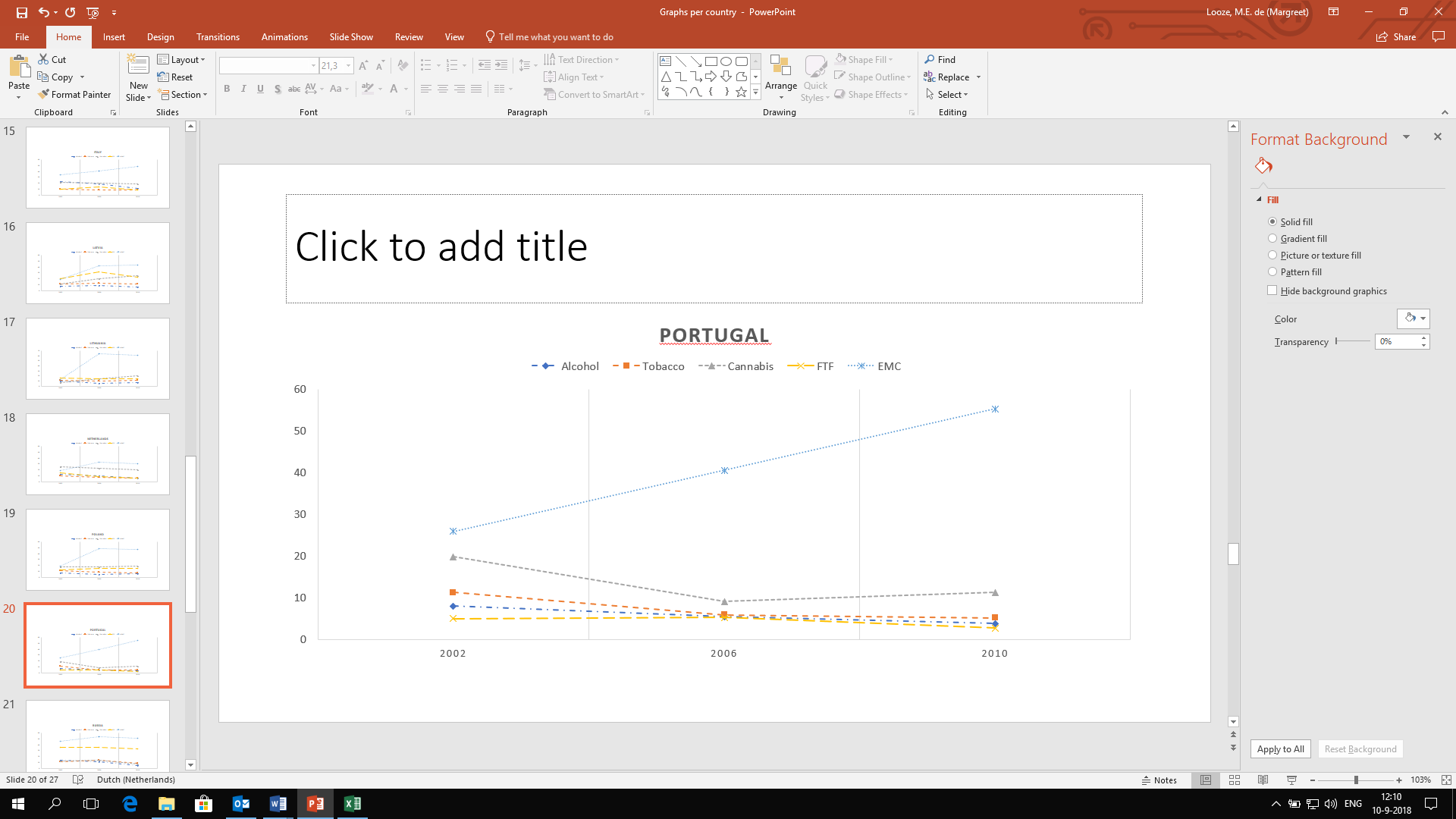

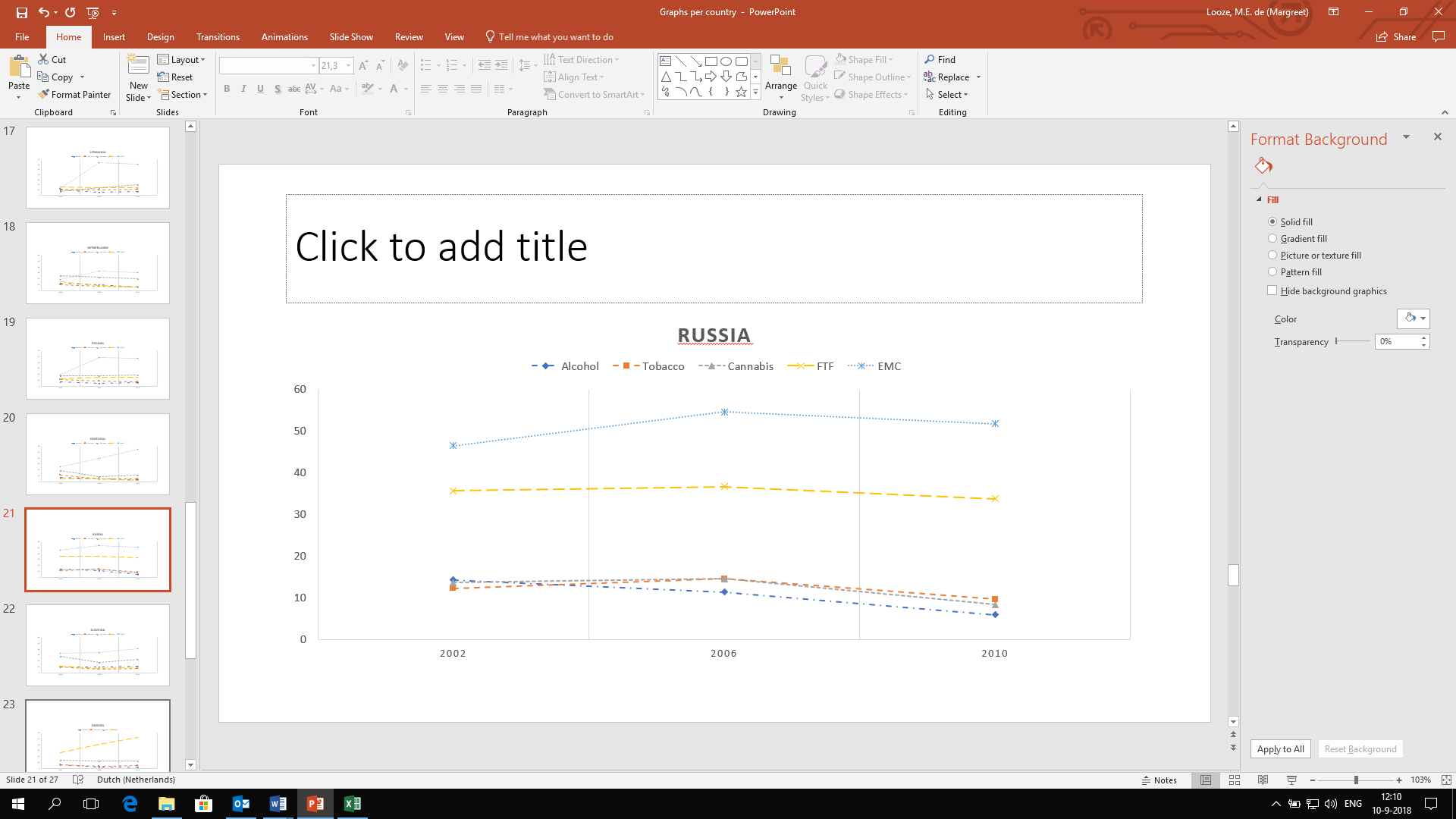


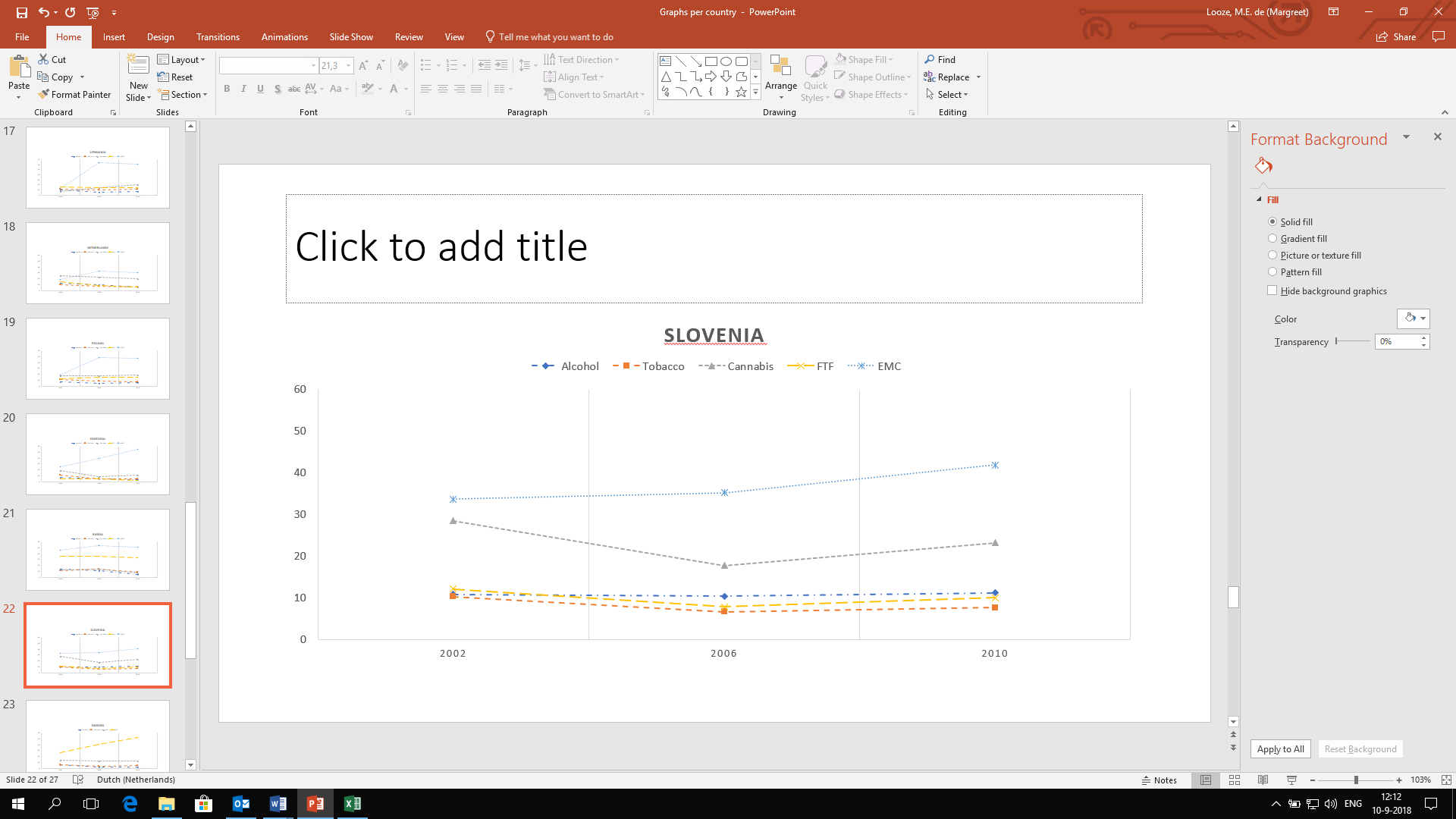

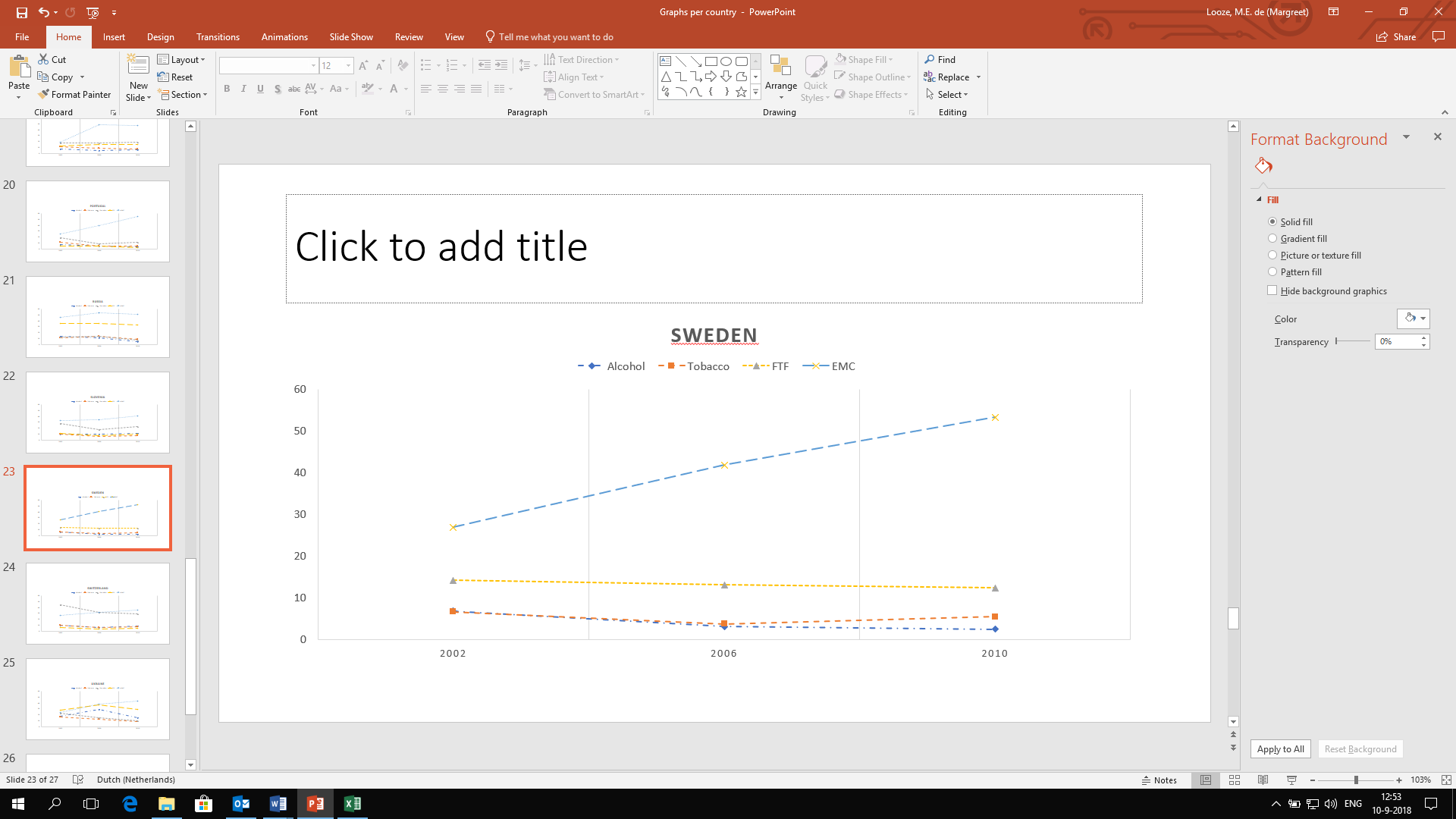


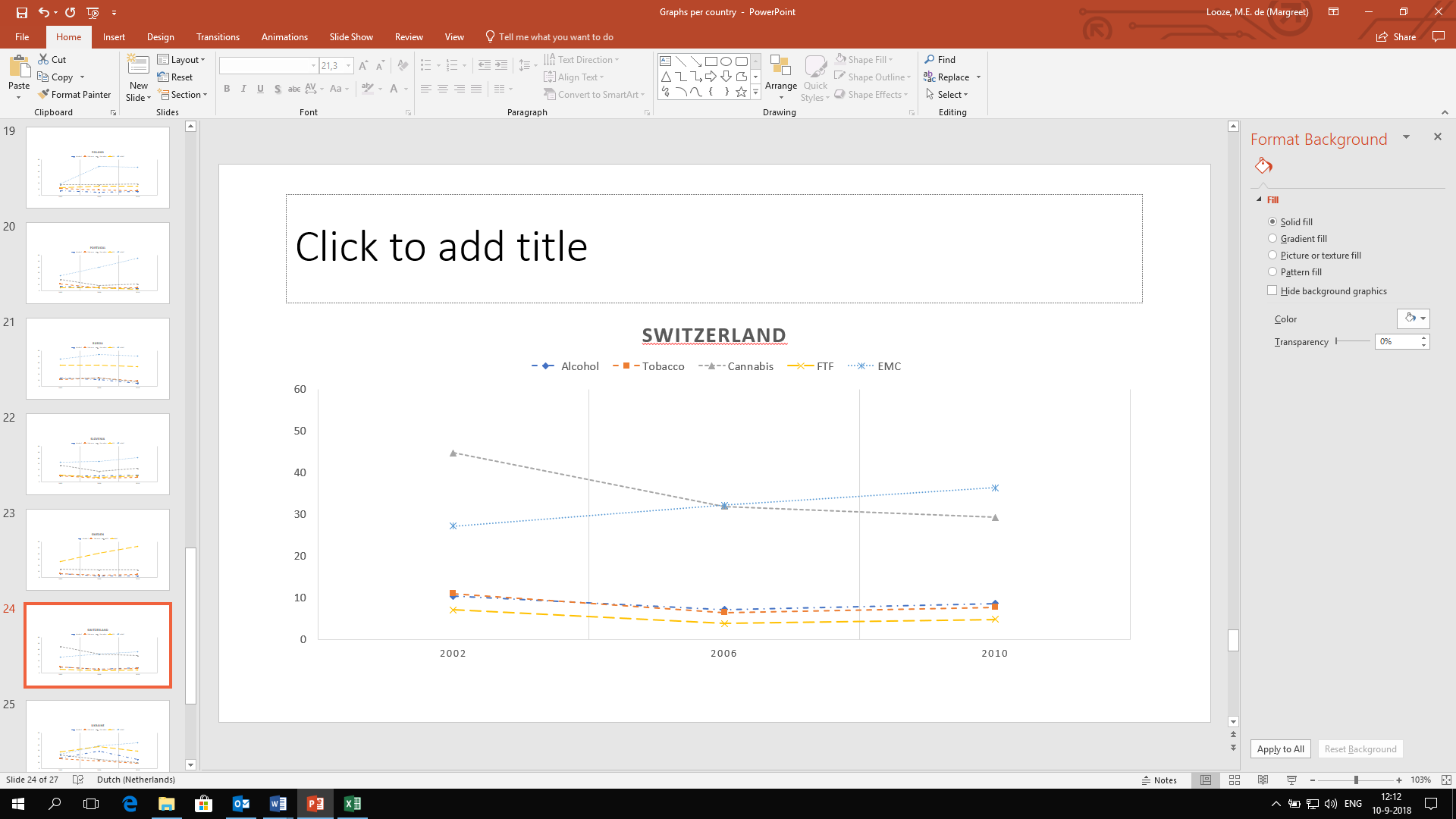

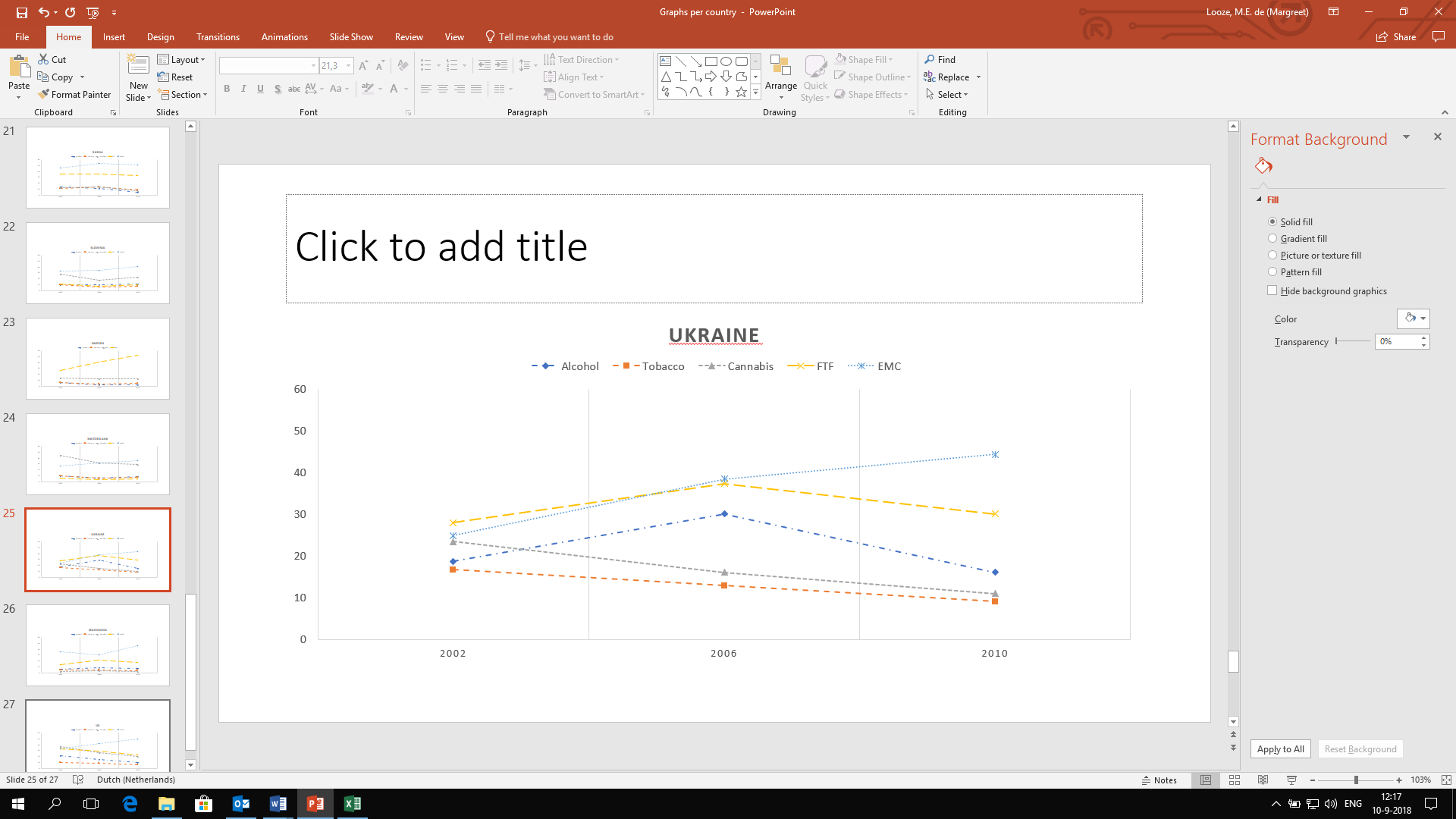


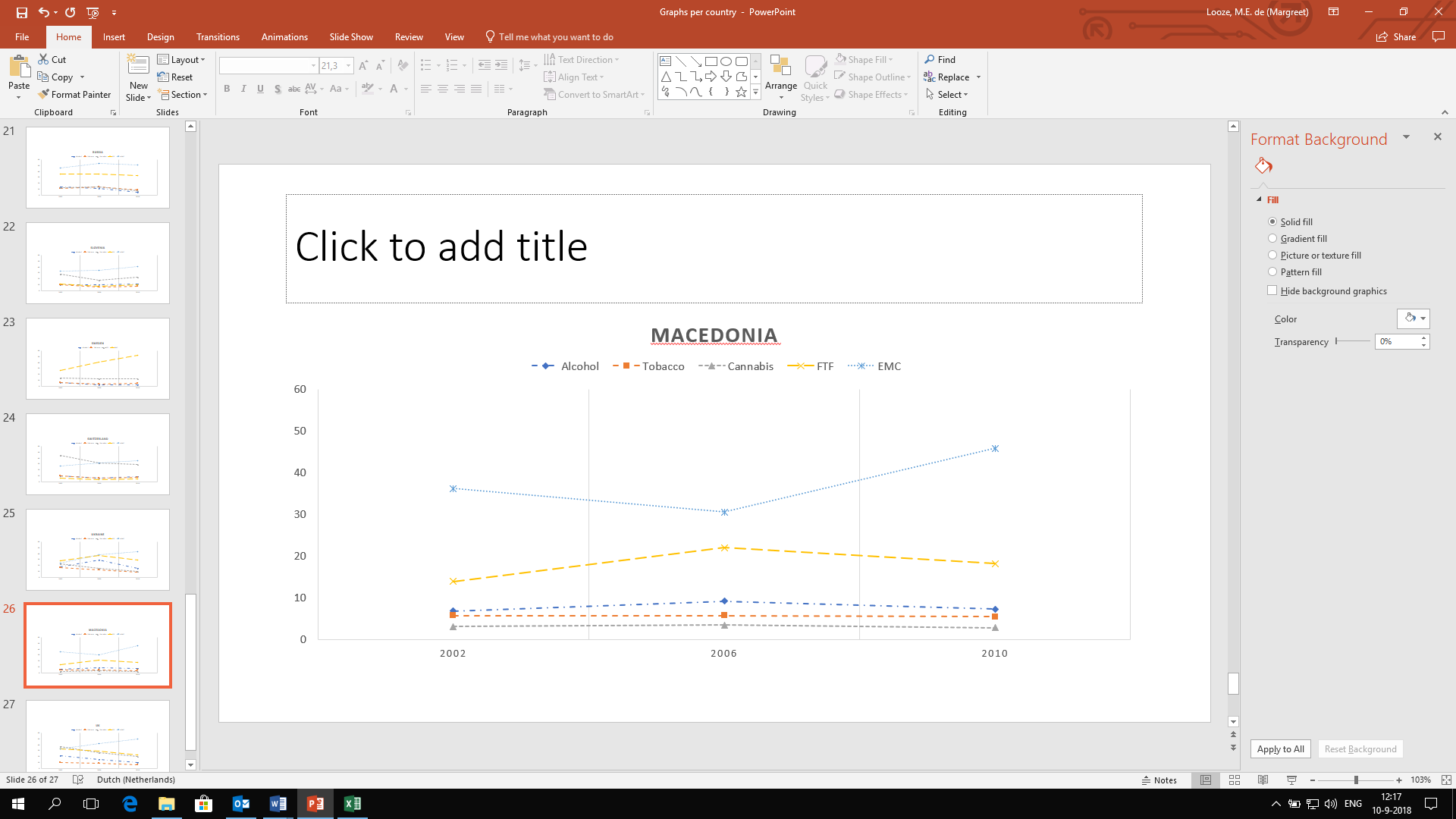

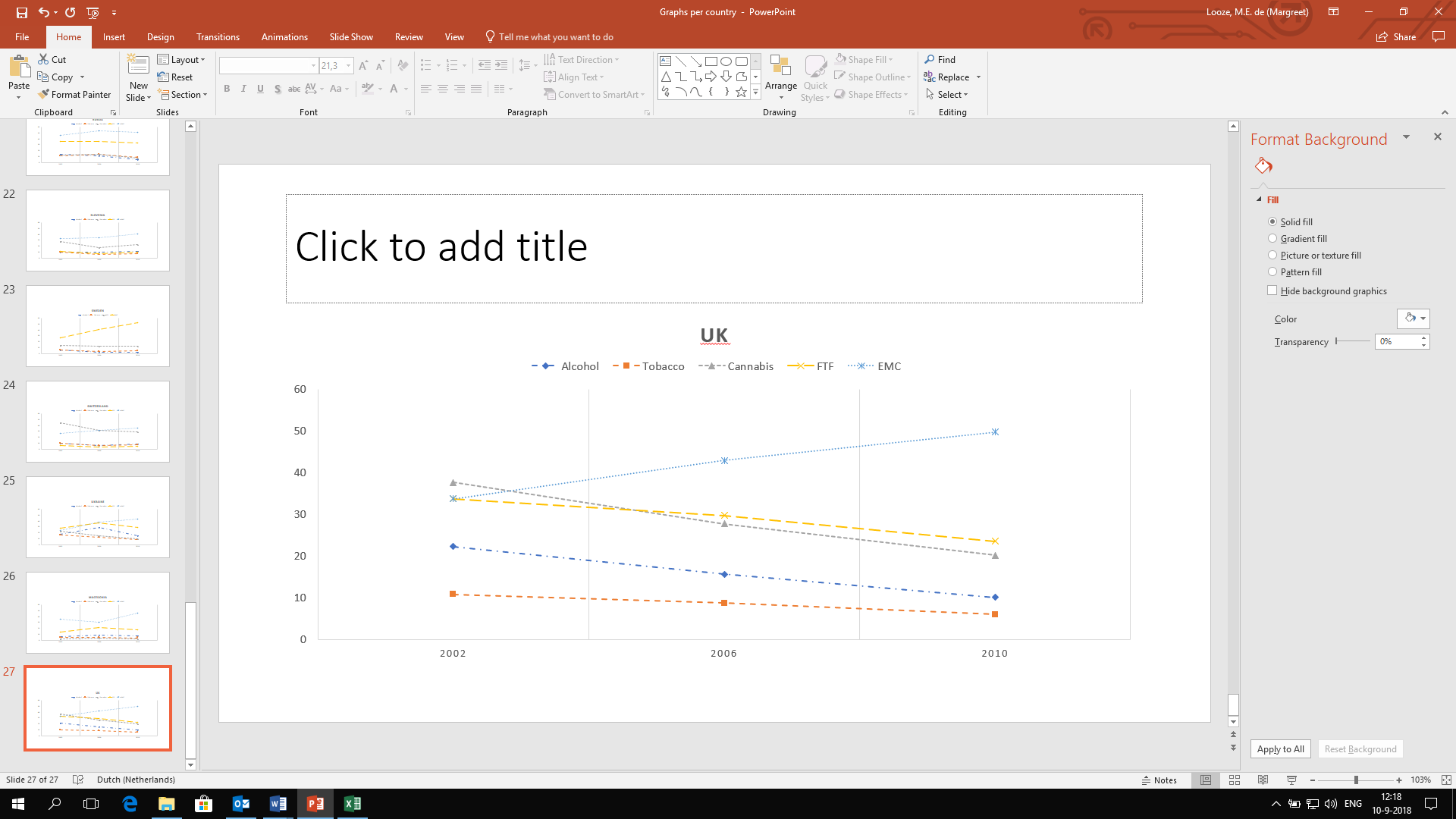


Supplementary Figure 1a-z. Graphs presenting trends over time (2002-2010) in substance use, face-to-face contact with peers in the evening, and electronic media communication, per country.

*Note.* Alcohol = weekly alcohol use; Tobacco = weekly smoking; Cannabis = lifetime cannabis use; FTF = daily face-to-face contact with peers in the evening; EMC = daily electronic media communication.

Swedish trend data on cannabis use were not available.
